# Mutual Regulation of Epicardial Adipose Tissue and Myocardial Redox State by PPAR-γ/Adiponectin Signalling

**DOI:** 10.1161/CIRCRESAHA.115.307856

**Published:** 2016-03-03

**Authors:** Alexios S. Antonopoulos, Marios Margaritis, Sander Verheule, Alice Recalde, Fabio Sanna, Laura Herdman, Costas Psarros, Hussein Nasrallah, Patricia Coutinho, Ioannis Akoumianakis, Alison C. Brewer, Rana Sayeed, George Krasopoulos, Mario Petrou, Akansha Tarun, Dimitris Tousoulis, Ajay M. Shah, Barbara Casadei, Keith M. Channon, Charalambos Antoniades

**Affiliations:** From the Division of Cardiovascular Medicine, Radcliffe Department of Medicine, University of Oxford, Oxford, United Kingdom (A.S.A., M.M., A.R., F.S., L.H., C.P., P.C., I.A., A.T., B.C., K.M.C., C.A.); Cardiac Electrophysiology Group, Department of Physiology, Maastricht University, Maastricht, The Netherlands (S.V., H.N.); Department of Cardiology, Athens University Medical School, Athens, Greece (D.T.); Cardiovascular Division, King’s College London BHF Centre, London, United Kingdom (A.C.B., A.M.S.); and Department of Cardiac Surgery, John Radcliffe Hospital, Oxford, United Kingdom (R.S., G.K., M.P.).

**Keywords:** adiponectin, adipose tissue, myocardium, obesity, oxidative stress

## Abstract

**Rationale:**

Adiponectin has anti-inflammatory effects in experimental models, but its role in the regulation of myocardial redox state in humans is unknown. Although adiponectin is released from epicardial adipose tissue (EpAT), it is unclear whether it exerts any paracrine effects on the human myocardium.

**Objective:**

To explore the cross talk between EpAT-derived adiponectin and myocardial redox state in the human heart.

**Methods and Results:**

EpAT and atrial myocardium were obtained from 306 patients undergoing coronary artery bypass grafting. Functional genetic polymorphisms that increase *ADIPOQ* expression (encoding adiponectin) led to reduced myocardial nicotinamide adenine dinucleotide phosphate oxidase–derived O_2_^−^, whereas circulating adiponectin and *ADIPOQ* expression in EpAT were associated with elevated myocardial O_2_^−^. In human atrial tissue, we demonstrated that adiponectin suppresses myocardial nicotinamide adenine dinucleotide phosphate oxidase activity, by preventing AMP kinase–mediated translocation of Rac1 and p47^phox^ from the cytosol to the membranes. Induction of O_2_^−^ production in H9C2 cardiac myocytes led to the release of a transferable factor able to induce peroxisome proliferator-activated receptor-γ–mediated upregulation of *ADIPOQ* expression in cocultured EpAT. Using a *NOX2* transgenic mouse and a pig model of rapid atrial pacing, we found that oxidation products (such as 4-hydroxynonenal) released from the heart trigger peroxisome proliferator-activated receptor-γ–mediated upregulation of *ADIPOQ* in EpAT.

**Conclusions:**

We demonstrate for the first time in humans that adiponectin directly decreases myocardial nicotinamide adenine dinucleotide phosphate oxidase activity via endocrine or paracrine effects. Adiponectin expression in EpAT is controlled by paracrine effects of oxidation products released from the heart. These effects constitute a novel defense mechanism of the heart against myocardial oxidative stress.

## Abstract 2

Supplemental Digital Content is available in the text.

Dysregulation of myocardial redox signaling is involved in the pathophysiology of multiple cardiac diseases.^1^ Nicotinamide adenine dinucleotide phosphate (NADPH) oxidases are major enzymatic sources of reactive oxygen species in the heart^2^ and have been linked in the past to cardiac pathologies such as atrial fibrillation,^3–5^ myocardial hypertrophy,^6^ heart failure,^7^ and others.^8^ Metabolic abnormalities such as obesity or diabetes mellitus are associated with increased NADPH oxidases activity in the cardiovascular system^8,9^ although the underlying mechanisms of these links are controversial.^10,11^ Because pharmacological treatments able to suppress myocardial NADPH oxidases (eg, statins as a part of their pleiotropic effects in the human heart^5^) have failed to prevent cardiac disease progression in humans,^12^ better understanding of the endogenous mechanisms regulating NADPH oxidases in the human heart is essential for the development of new therapeutic strategies to target myocardial redox state.^13^

Adipose tissue affects the cardiovascular system by secreting a wide range of bioactive products known as adipokines.^14^ Adiponectin is an important adipokine with anti-inflammatory^15^ and antioxidant effects on the vasculature^16^ and the heart of experimental models,^17^ but its role in the regulation of myocardial redox state in humans is unknown.

In healthy individuals, low circulating adiponectin is associated with increased cardiovascular risk^18^; however, in individuals with ischemic heart disease (IHD), adiponectin gene expression in adipose tissue is increased^14^ and high circulating adiponectin predicts adverse clinical outcome.^19^ Importantly, given that the human heart is surrounded by biologically active epicardial adipose tissue (EpAT), adiponectin released from it may exert additional paracrine effects on the underlying myocardium in a similar way perivascular adipose tissue exerts paracrine effects on the vascular wall,^20^ a concept that has not been previously explored.

In this study, we explore the role of adiponectin in the regulation of myocardial redox state in patients with IHD, and we characterize the underlying molecular mechanisms mediating adiponectin’s effects on the heart. In addition, we define the mechanisms controlling peroxisome proliferator-activated receptor (PPAR)-γ/adiponectin signaling in human EpAT, and we introduce the novel concept of an inside to outside signaling released from the human heart in conditions of increased myocardial oxidative stress, which controls the biosynthetic activity of the neighboring EpAT.

## Methods

### Study Population

The study population consisted of 306 patients undergoing coronary artery bypass grafting surgery; 247 of these were included in the Clinical Associations Studies and 59 into the ex vivo arm of the study. Blood samples were obtained on the morning of surgery, whereas samples of myocardium and adipose tissue were collected during surgery, as described below. Myocardial and adipose tissue samples were used for ex vivo experiments to address the mechanisms regulating the cross talk between EpAT and human myocardium as described below. Exclusion criteria were any inflammatory, infectious, liver or renal disease, or malignancy. Patients receiving nonsteroidal anti-inflammatory drugs, dietary supplements, or antioxidant vitamins were also excluded.

### Design of the Clinical Associations Studies

In 247 patients undergoing coronary artery bypass grafting, blood samples were collected at the morning of surgery for measuring circulating adiponectin and other biomarkers as well as for DNA extraction and genotyping. Samples of right atrial appendage obtained from the cannulation site^5^ were used to quantify NADPH oxidase–derived superoxide anions (O_2_^−^), aiming to relate it with circulating adiponectin and the expression of *ADIPOQ* gene (encoding adiponectin) in different adipose tissue depots. These myocardial samples were also used to study the myocardial expression of *ADIPOQ* and adiponectin receptors. In addition, EpAT from the atrioventricular groove and thoracic adipose tissue (ThAT) from the outer surface of the pericardium^14^ were collected and used to quantify the expression of *ADIPOQ* and other target genes, aiming to search for predictors of myocardial redox state as described below. More detail on samples collection and processing is provided in the Online Data Supplement.

### Blood Sampling and Measurement of Circulating Biomarkers

Venous blood samples were obtained after 8 hours of fasting, on the morning of the operation, and used for measurement of circulating biomarkers (as described in the Online Data Supplement).

### DNA Extraction and Genotyping

Genomic DNA extraction from whole blood and genotyping were performed using standard methods (as described in the Online Data Supplement).

### Myocardial Superoxide Measurements

Myocardial O_2_^−^ production was measured in right atrial appendage samples using lucigenin (5 μmol/L)–enhanced chemiluminescence (as described in the Online Data Supplement).

### RNA Isolation and Quantitative Real-Time-Polymerase Chain Reaction

Samples of adipose tissue and right atrial appendage were used for RNA extraction and gene expression studies (as described in the Online Data Supplement).

### Ex Vivo Experiments With Human Myocardium

To examine the direct effects of adiponectin on myocardial O_2_^−^ production, human myocardial tissue from the right atrial appendage was incubated ex vivo for 2 hours with/without adiponectin in the presence/absence of the pharmacological inhibitor of AMP-kinase (AMPK), compound C (CC). Briefly, myocardial tissue was washed in ice-cold Krebs HEPES buffer, and then cut into thin strips containing all myocardial layers. The tissue was first equilibrated for 20 minutes in Krebs HEPES buffer pH 7.35 at 37°C, and then incubated for 2 hours in the presence or absence of recombinant full-length adiponectin 0.3 μmol/L (10 μg/mL; BioVendor)±CC (10 μmol/L). The effect of adiponectin on myocardial O_2_^−^ (basal and NADPH-stimulated O_2_^−^) was quantified by lucigenin (5 μmol/L)–enhanced chemiluminescence (as described in the Online Data Supplement ). To estimate the effect of adiponectin on NADPH oxidase–derived O_2_^−^,we used the pan-NADPH oxidase inhibitor Vas2870^21^ (40 µmol/L; Sigma Aldrich).

### Ex Vivo Experiments With Human Adipose Tissue

Samples of ThAT and EpAT obtained from patients in the ex vivo study arm were used to estimate the effects of lipid oxidation products on *ADIPOQ* gene expression in an ex vivo bioassay. Briefly, adipose tissue was isolated and washed in sterile phosphate buffer saline (PBS). The samples of adipose tissue of each type were transferred to the laboratory within 30 minutes from harvesting. The samples were then cut into ≈1- to 2-mm^3^ cubes, washed, and equilibrated for 2 hours at 37°C in Medium-199 containing HEPES (25 mmol/L), gentamycin 105 μmol/L (50 μg/mL), and fatty acid–free BSA (1%), in the presence of protease inhibitor (Roche Applied Science) in a cell culture incubator with 5% CO_2_ atmosphere. At the end of the equilibration period, the media was replaced by fresh media (1 mL/200 mg tissue) and incubated for 16 hours as above but in the presence or absence of (1) H_2_O_2_ (100 μmol/L), (2) malonyldialdehyde (1 mmol/L), (3) 4-hydroxynonenal (HNE, 30 μmol/L)±T0070907 (10 μmol/L, an inhibitor of PPAR-γ). For the samples treated with T0070907, this agent was also used during the equilibration period. At the end of the 16-hour incubation period, AT samples were filtered, collected, and stored at −80°C until analysis. Quantitative real-time-polymerase chain reactions were performed (as described in the Online Data Supplement) to determine *ADIPOQ*, *PPAR-γ*, and *CD36* gene expression.

### Cocultures of Rat EpAT With H9C2 Cells

To evaluate the interaction between AT and myocardium, the rat cardiac myocyte–derived cell line H9c2 was coincubated with rat EpAT ex vivo. Briefly, H9c2 cells were differentiated to cardiac myocytes in Dulbecco’s Modified Eagle Medium (Sigma-Aldrich) supplemented with 1% horse serum (Sigma-Aldrich). The cells were exposed to either NADPH (100 μmol/L) or phorbol-12-myristate-13-acetate (PMA; 160 nmol/L) for 2 hours as a means to induce O_2_^−^ generation from NADPH oxidases. Freshly collected rat EpAT from female Wistar rats was then added to the culture medium and coincubated with H9c2-derived cardiac myocytes with or without NADPH (100 μmol/L; n=7) or PMA 160 nmol/L (n=7) for 16 hours. To control for direct effects of NADPH or PMA on adipose tissue, EpAT was also incubated alone in the presence or absence of NADPH (100 μmol/L) or PMA (160 nmol/L). To prevent any direct effects of endogenous O_2_^−^ in EpAT, additional interventions with NADPH (100 μmol/L) and polyethylene glycol (PEG)-SOD (300 U/mL, a scavenger of O_2_^−^) or Vas2870 (10 nmol/L, an inhibitor of NADPH oxidases) were included. At the end of the incubation period, EpAT was collected for gene expression studies (as described in the Online Data Supplement).

### Measurement of Intracellular NADP/NADPH Levels

Intracellular NADP/NADPH levels were measured using a commercially available fluorometric assay (Abcam kit, Cambridge, United Kingdom) as described in the Online Data Supplement.

### Western Blots in Human Myocardial Samples

Western blotting was performed as described in the Online Data Supplement.

### Measurement of Myocardial Rac1 Activation and Membrane Translocation Experiments

Rac1 activation and membrane translocation of Rac1 and p47^phox^ were determined in right atrial appendage samples, as previously described^22^ (Online Data Supplement).

### Animal Studies

#### Mouse Model

Cardiac myocyte–specific *NOX2*-transgenic mice^23^ (*mNOX2-tg*; Online Data Supplement) were used as a model of chronically increased myocardial oxidative stress to test the impact of increased myocardial Nox2-derived O_2_^−^ production on adiponectin expression in subcutaneous and pericardial adipose tissue (attached to the apex of the heart).

Twenty-week-old transgenic male mice and wild-type littermate controls were euthanized, and whole heart samples and samples of subcutaneous and pericardial AT were harvested and studied.

#### Pig Model

Given the limitations related to the study of pericardial AT in mice (limited amount of tissue, not always present), we used a larger mammal model (pig), whose EpAT is directly attached on the heart muscle, mimicking the interaction between myocardium and EpAT in humans. To induce a chronic increase in oxidative stress in the atrial myocardium,^4^ we used a standardized protocol of rapid atrial pacing for 4 weeks and collected EpAT from the posterior left atrium at the end of this period. Subcutaneous AT was used as a control depot. More details on the pig model are provided in the Online Data Supplement.

Myocardial tissue samples from the 2 animal models were homogenized and used for lucigenin-enhanced chemiluminescence experiments to assess myocardial NADPH oxidases activity (as described in the Online Data Supplement) and to blot for 4HNE and malonyldialdehyde protein adducts (antibodies by Abcam). RNA was extracted from myocardial and AT samples and used for gene expression studies (as described in the Online Data Supplement).

### Statistical Analysis

Continuous variables were tested for normal distribution using Kolmogorov–Smirnov test. Non-normally distributed variables were log-transformed for analysis. In the Clinical Associations Study arm, continuous variables among 3 groups were compared using 1-way ANOVA followed by the Bonferroni post hoc test for individual comparisons, whereas comparisons between 2 groups were performed by unpaired *t* tests. Categorical variables were compared using the χ^2^ test as appropriate. Correlations between continuous variables were assessed by bivariate analysis, and the Pearson coefficient was estimated.

For the ex vivo experiments (in which serial segments from the same right atrial appendage were incubated with multiple interventions), we performed repeated-measures ANOVA and paired *t* tests for individual comparisons, followed by the Bonferroni post hoc correction for multiple testing as appropriate. In the Clinical Associations study arm, correlations between continuous variables were tested by calculating the Pearson correlation coefficient. Multivariable linear regression was performed by using log(NADPH-stimulated O_2_^-^) or log(serum adiponectin) as dependent variables and by using demographic/biological variables that showed a significant association with the dependent variable in univariate analysis at the level of 15% as independent variables.

For the animal studies, comparisons of *mNOX2*-tg versus wild-type mice or rapid atrial pacing versus sham were performed using unpaired *t* test of the log-transformed values for the respective variables. Power calculations are provided in the Online Data Supplement. All statistical tests were performed with SPSS version 20.0, and values of *P*<0.05 were considered statistically significant.

## Results

### Interactions Between Adiponectin and Myocardial NADPH Oxidases Activity in Patients With IHD

**Figure 1.**
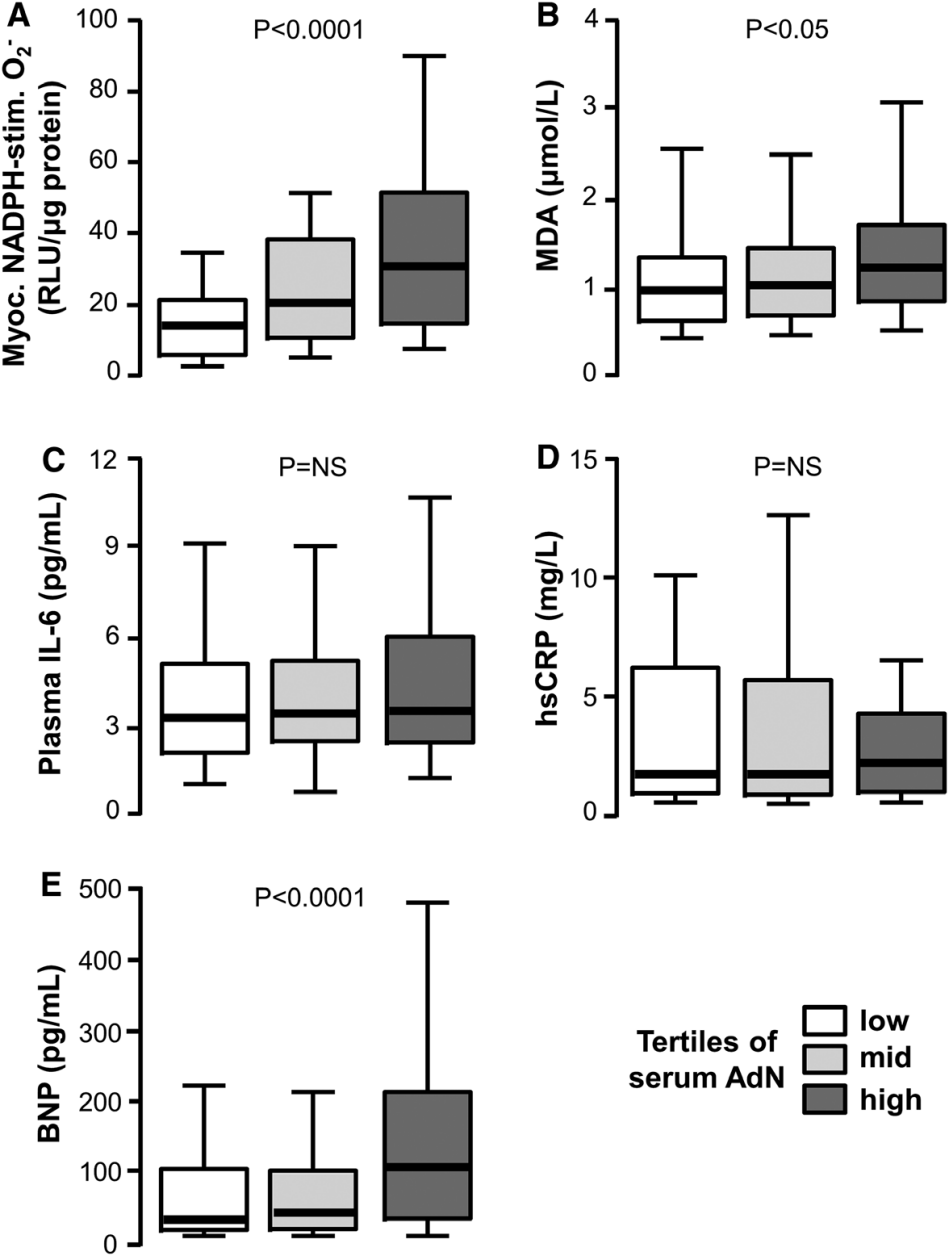
**Circulating adiponectin (AdN) is positively related with myocardial redox state in patients with ischemic heart disease.** In the Clinical Associations Studies, high circulating AdN levels were paradoxically related with high myocardial nicotinamide adenine dinucleotide phosphate (NADPH)–stimulated superoxide (O_2_^−^) (**A**) and high plasma malonyldialdehyde (MDA; a marker of systemic oxidative stress; **B**). There was no association of circulating adiponectin with plasma interleukin-6 (IL-6; **C**) or high-sensitivity C-reactive protein (hsCRP; **D**). High circulating adiponectin was also positively related with high plasma brain natriuretic peptide (BNP; **E**). Values are expressed as median (25th–75th percentile).

The characteristics of the patients included in the Clinical Associations Studies are presented in the Table. In this cohort of patients with IHD, we first explored the association between adiponectin levels and myocardial redox state. We observed a positive association between circulating adiponectin and myocardial NADPH-stimulated O_2_^−^ (Figure 1A), as well as between circulating adiponectin and malonyldialdehyde (Figure 1B), a plasma marker of oxidative stress. However, there was no significant association between adiponectin and either interleukin-6 (Figure 1C) or high-sensitivity C-reactive protein (Figure 1D), suggesting that systemic inflammation does not confound the association between circulating adiponectin and myocardial NADPH oxidases activity. We also observed a significant association between plasma brain natriuretic peptide (BNP) and circulating adiponectin (Figure 1E), but not between BNP and NADPH-stimulated O_2_^−^ in the myocardium (Online Table I) or plasma malonyldialdehyde (*r*=0.147; *P*=0.092). To further explore the association between myocardial redox state and serum adiponectin, we performed univariate analysis followed by multivariable analysis searching for predictors of myocardial NADPH oxidases-derived O_2_^−^ (Online Table I), confirming that the correlation between circulating adiponectin and myocardial NADPH oxidases activity is independent of BNP and other potential confounders (Figure 1A; Online Table I). In addition, this was independent of medication such as statins (βst=−0.314; *P*=0.0001), antiplatelet treatment (βst=−0.179; *P*=−0.29), β-blockers (βst=0.013; *P*=0.877), calcium channel blockers (βst=−0.019; *P*=0.807), or angiotensin-converting enzyme inhibitor/ angiotensin receptor blocker (βst=0.016; *P*=0.833), with *R*^2^ for the model 0.238. Interestingly, serum levels of adiponectin or *ADIPOQ* gene expression in EpAT were not related with the incidence of postoperative atrial fibrillation (data not shown), which we have previously shown to be increased in patients with high atrial NADPH-oxidases activity.^5^

**Table.**
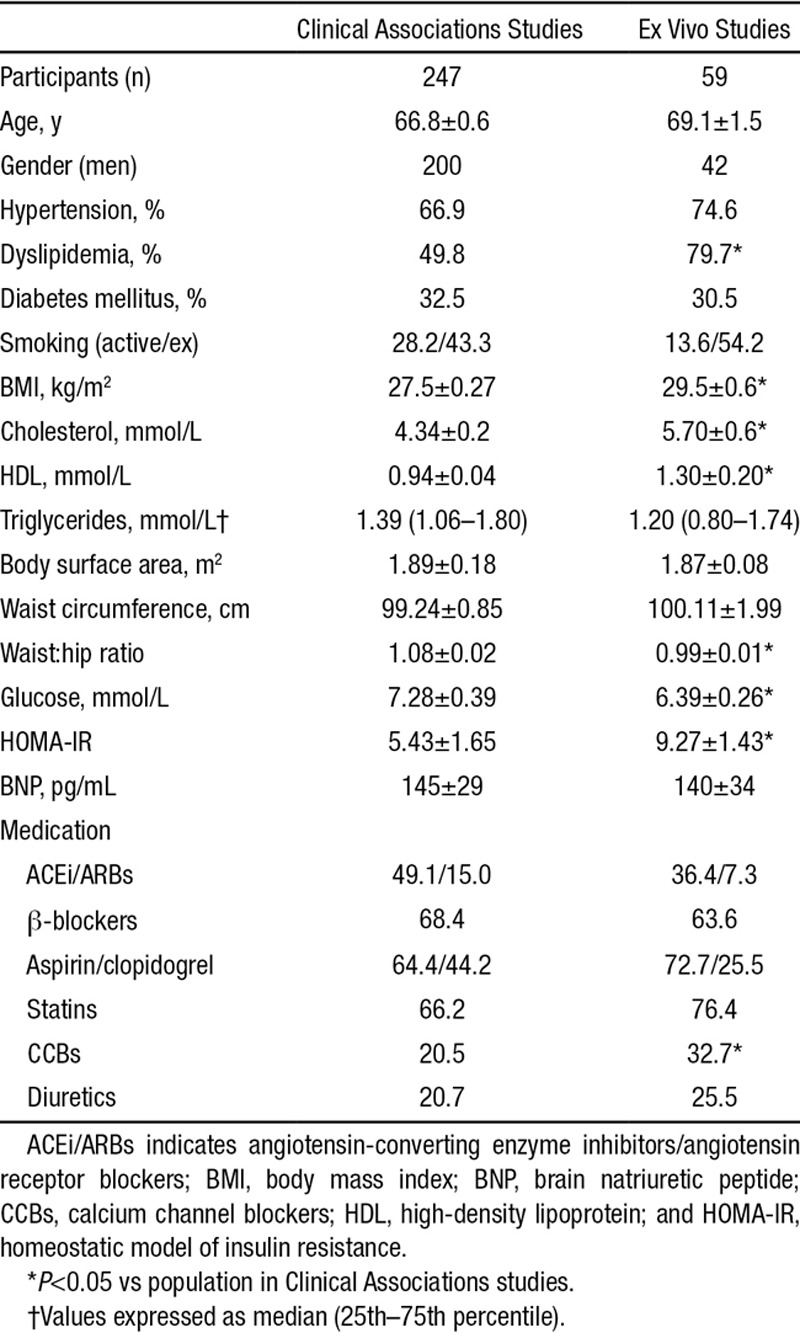
Demographic Characteristics of Study Participants

**Figure 2.**
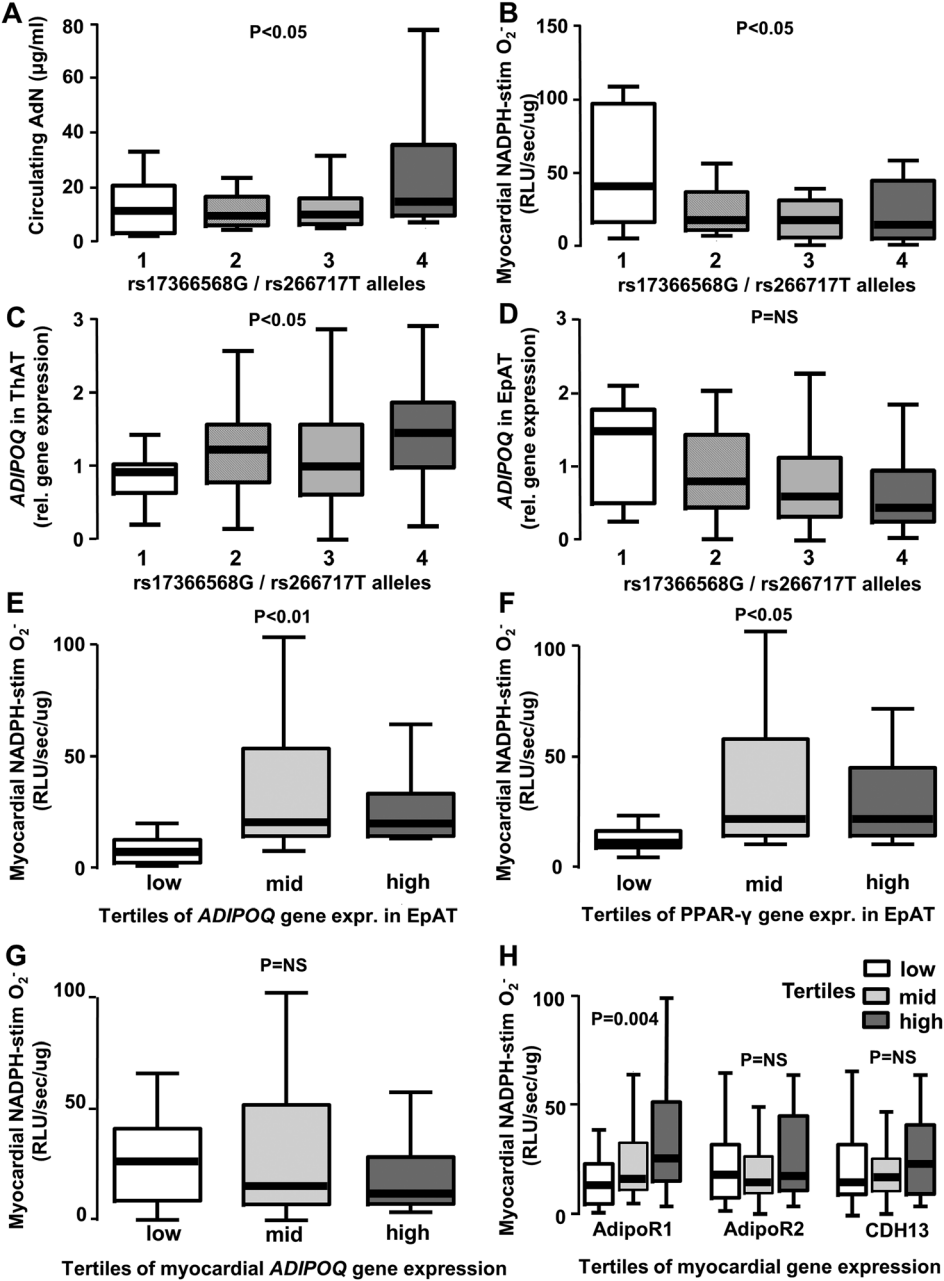
**Genetically conferred increases in adiponectin (AdN) bioavailability are causally associated with lower nicotinamide adenine dinucleotide phosphate (NADPH) oxidase activity in the human myocardium.** The total number of rs17366568G alleles (polymorphism in ADIPOQ gene) and rs266717T alleles (polymorphism in ADIPOQ gene promoter) had an additive effect on circulating AdN levels (**A**) and was associated with reduced NADPH–stimulated superoxide (O_2_^−^) in human myocardium (**B**). The number of rs17366568G/rs266717T alleles was positively associated with higher *ADIPOQ* gene expression in thoracic adipose tissue (ThAT, **C**), but not associated in epicardial adipose tissue (EpAT; **D**). Patients with higher *ADIPOQ* (**E**) or peroxisome proliferator-activated receptor (PPAR)-γ (**F**) gene expression in EpAT also had higher NADPH-stimulated O_2_^−^ production in their myocardium. Higher myocardial NADPH-stimulated O_2_^−^ was not associated with endogenous *ADIPOQ* gene expression in the heart (**G**), but was associated with higher gene expression of adiponectin receptor-1 (AdipoR1), but not of AdipoR2 or T-cadherin (CDH13) in human myocardial tissue (**H**). Values are expressed as median (25th–75th percentile). RLU indicates relative light units.

To further explore the association between adiponectin and myocardial redox state, we genotyped our study population for 2 functional single nucleotide polymorphisms in the *ADIPOQ* locus encoding adiponectin (both single nucleotide polymorphisms with known effect on adiponectin levels), located in the *ADIPOQ* locus (rs266717) and in the promoter region (rs17366568).^24,25^ We found that the number of rs266717G and rs17366568T alleles was positively associated with serum adiponectin (Figure 2A) and negatively associated with myocardial NADPH oxidases activity (Figure 2B). This implies that low adiponectin production in human AT is causally associated with higher myocardial NADPH oxidases activity. Furthermore, there was a significant effect of the single nucleotide polymorphisms on the expression of *ADIPOQ* gene in ThAT (Figure 2C), but this was not observed in EpAT (Figure 2D), suggesting that local mechanisms may over-ride the influence of genetic background on *ADIPOQ* gene expression in EpAT but not in remote adipose tissue depots such as ThAT.

We next examined whether obesity and adipose tissue distribution alter the regulation of *ADIPOQ* gene expression in different adipose tissue depots. We confirmed that *ADIPOQ* gene expression in ThAT was strongly inversely correlated with waist:hip ratio and body mass index, whereas *PPAR-γ* expression was similarly correlated with waist:hip ratio (although its association with body mass index was borderline significant; Online Figure IA–ID). However, these associations were not present in EpAT (Online Figure IE–IH). These discordant findings between EpAT and ThAT suggest that PPAR-γ/adiponectin signaling in EpAT is controlled by local mechanisms, possibly originating in the heart, rather than by systemic effects related to obesity and insulin resistance.

Importantly, there was a positive association between myocardial NADPH-stimulated O_2_^−^ and the expression of both *ADIPOQ* (Figure 2E) and PPAR-γ genes (Figure 2F) in EpAT, suggesting that increased myocardial O_2_^−^ may influence the expression of *ADIPOQ* in EpAT, possibly by regulating PPAR-γ expression. *ADIPOQ* gene expression is known to be partly under the control of PPAR-γ signaling. This was confirmed by the association between log(PPAR-γ) and log(*ADIPOQ*) gene expression in EpAT of patients with IHD (*r*=0.598; *P*<0.0001).

To explore whether myocardial resistance to adiponectin is responsible for the positive association between myocardial redox state and adiponectin levels (systemic and EpAT expression), we studied the expression of *ADIPOQ* and adiponectin receptors (*ADIPOR1, ADIPOR2*, and *CDH13*) in the myocardium of patients with advanced coronary atherosclerosis. There was no association between myocardial NADPH oxidases activity and *ADIPOQ* gene expression (Figure 2G), whereas there was a positive association between myocardial NADPH oxidase activity and *ADIPOR1* (but not *ADIPOR2* or *CDH13*; Figure 2H), suggesting that in the presence of increased myocardial reactive oxygen species generation, there is upregulation of *ADIPOR1* in human myocardium and an upregulation of *ADIPOQ* gene in EpAT.

### Adiponectin Directly Decreases NADPH Oxidases Activity in the Human Myocardium

**Figure 3.**
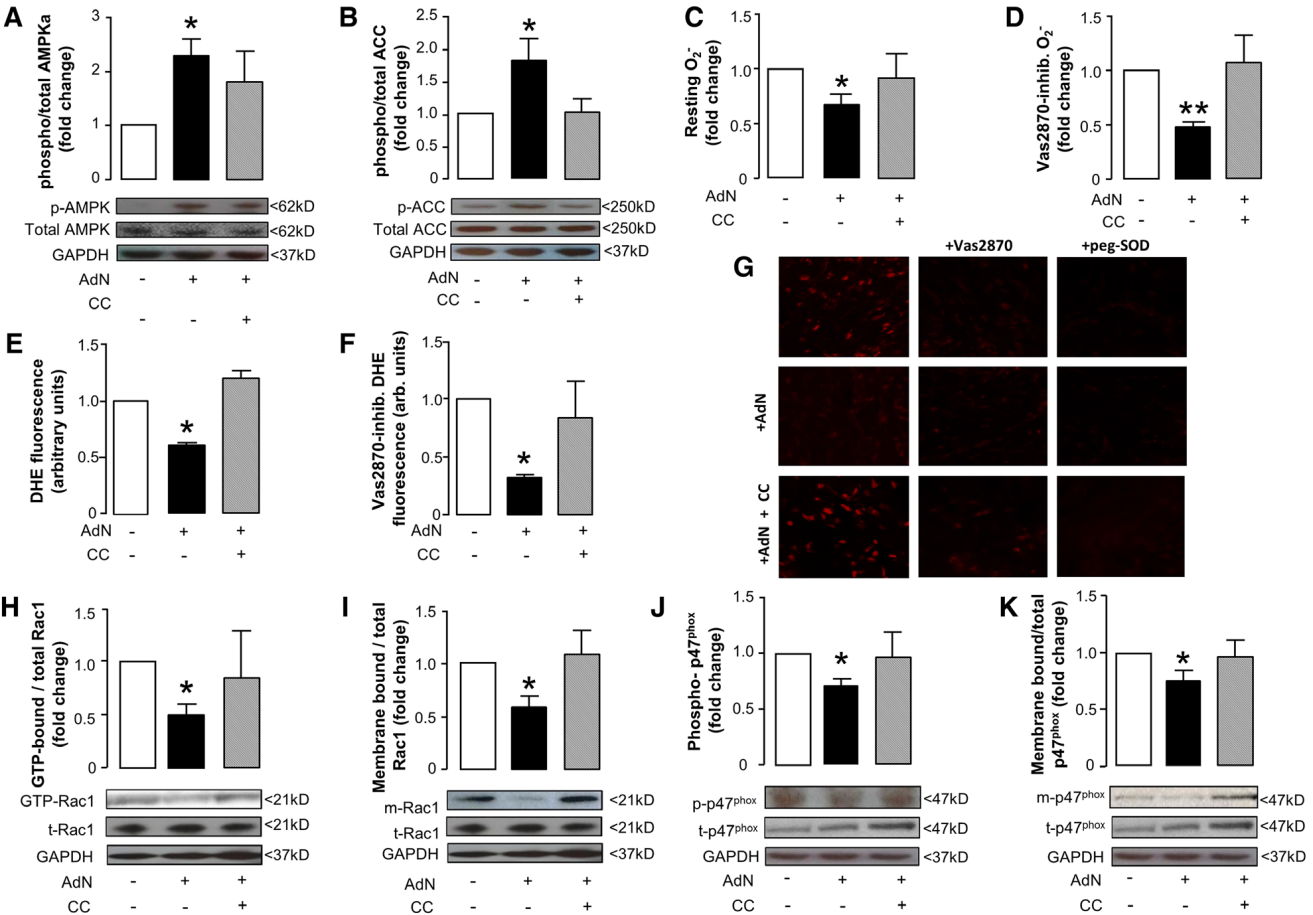
**Effects of recombinant adiponectin (AdN) on myocardial redox state in humans.**Ex vivo incubation of human myocardium with AdN (10 μg/mL) for 2 h resulted in increased phosphorylation of AMP-kinase (AMPK)-α at Thr172 (p-AMPK; **A**) leading to AMPK activation as assessed by the phosphorylation status of its downstream target acetyl-CoA carboxylase (ACC) at Ser79 (p-ACC; **B**), an effect reversed by compound C (CC; 10 μmol/L; **A** and **B**). AdN reduced superoxide (O_2_^−^) production in human myocardium (**C**) and specifically nicotinamide adenine dinucleotide phosphate (NADPH) oxidase activity, as assessed by measuring the Vas2870-inhibitable (40 μmol/L) O_2_^−^ (**D**); both effects were reversed by CC (**C** and **D**). These effects of AdN on myocardial O_2_^−^ were also confirmed by dihydroethidium (DHE) staining; AdN reduced both total and Vas2870-inhibitable DHE fluorescence, and these effects were reversed by CC (**E**–**G**). Importantly, AdN prevented Rac1 activation (assessed by measuring the ratio of GTP-Rac1:total Rac1 [t-Rac1; **H**]) and reduced the membrane-bound fraction of Rac1 (m-Rac1; **I**); both effects were reversed by CC. Similarly, AdN prevented p47^phox^ phosphorylation at its activatory site Ser359 (p-p47^phox^, **J**) and reduced the membrane-bound fraction of nicotinamide adenine dinucleotide phosphate (NADPH) oxidase subunit p47^phox^ (m-p47^phox^; **K**) in an AMPK-dependent manner (as both effects were reversed by CC; **J** and **K**). **A** and **B**, AdN: n=7 to 13 per group; CC: 4 to 7 per group; (**C**, **D**, **H**, **J**, **K**) AdN, n=7 to 12; CC group, n=4 to 6; (**E**–**I**) n=3 to 4 per group. Values are expressed as fold change vs control group and shown as mean±SEM; *P<0.05, **P<0.01 vs control.

To examine whether adiponectin has the ability to affect myocardial redox state in humans, myocardial tissue from patients with IHD (Table) was incubated with adiponectin (10 μg/mL) for 2 hours. Exogenous adiponectin rapidly induced phosphorylation of AMPK at the activatory site Thr172 (Figure 3A) and the downstream target acetyl-coA carboxylase, via phosphorylation at Ser79 (Figure 3B). As expected, the observed effects on acetyl-coA carboxylase phosphorylation, a marker of AMPK activity, were prevented by the AMPK inhibitor CC (Figure 3A and 3B). Similarly, adiponectin induced a rapid reduction of myocardial O_2_^−^ production that was reversed by CC (Figure 3C). Importantly, adiponectin suppressed the Vas2870-inhibitable fraction of myocardial O_2_^−^ in a CC-inhibitable manner (Figure 3D), suggesting that adiponectin inhibits NADPH oxidase activity via an AMPK-mediated mechanism. These AMPK-dependent effects of adiponectin were also confirmed in dihydroethidium staining experiments; adiponectin reduced both the total and the Vas2870-inhibitable dihydroethidium fluorescence (Figure 3E–3G). Adiponectin did not have any effects on the gene expression or protein levels of Nox isoforms or any NADPH oxidase subunits in human myocardium (Online Figure II), but prevented the activation of Rac1 (reduced the GTP-Rac1/total Rac1) and its membrane translocation (Figure 3H and 3I), as well as the phosphorylation of the p47^phox^ subunit of Nox2 at its activatory site Ser359 and its membrane translocation (Figure 3J and 3K). Both the effects of adiponectin on Rac1 and p47^phox^ were reversed by CC, suggesting that these effects involved AMPK signaling. Interestingly, the AMPK-mediated effects of adiponectin on Rac1 and p47^phox^ activation/membrane translocation were independent of any change in Akt activity, as evidenced by Western blotting for phospho-Akt at its activation site Ser473, or protein kinase C-α phosphorylation status (Online Figure II).

These data suggest that adiponectin suppresses NADPH oxidases activity in the human myocardium in an AMPK-dependent mechanism. Taken together with the observed positive association between myocardial NADPH oxidases activity and *ADIPOQ* gene expression in EpAT (Figure 2E), these findings suggest that myocardial O_2_^−^ production may be driving (directly or indirectly) the expression of PPAR-γ/*ADIPOQ* in EpAT.

### Identifying a Novel, Redox Sensitive Signal From Cardiac Myocytes to EpAT

**Figure 4.**
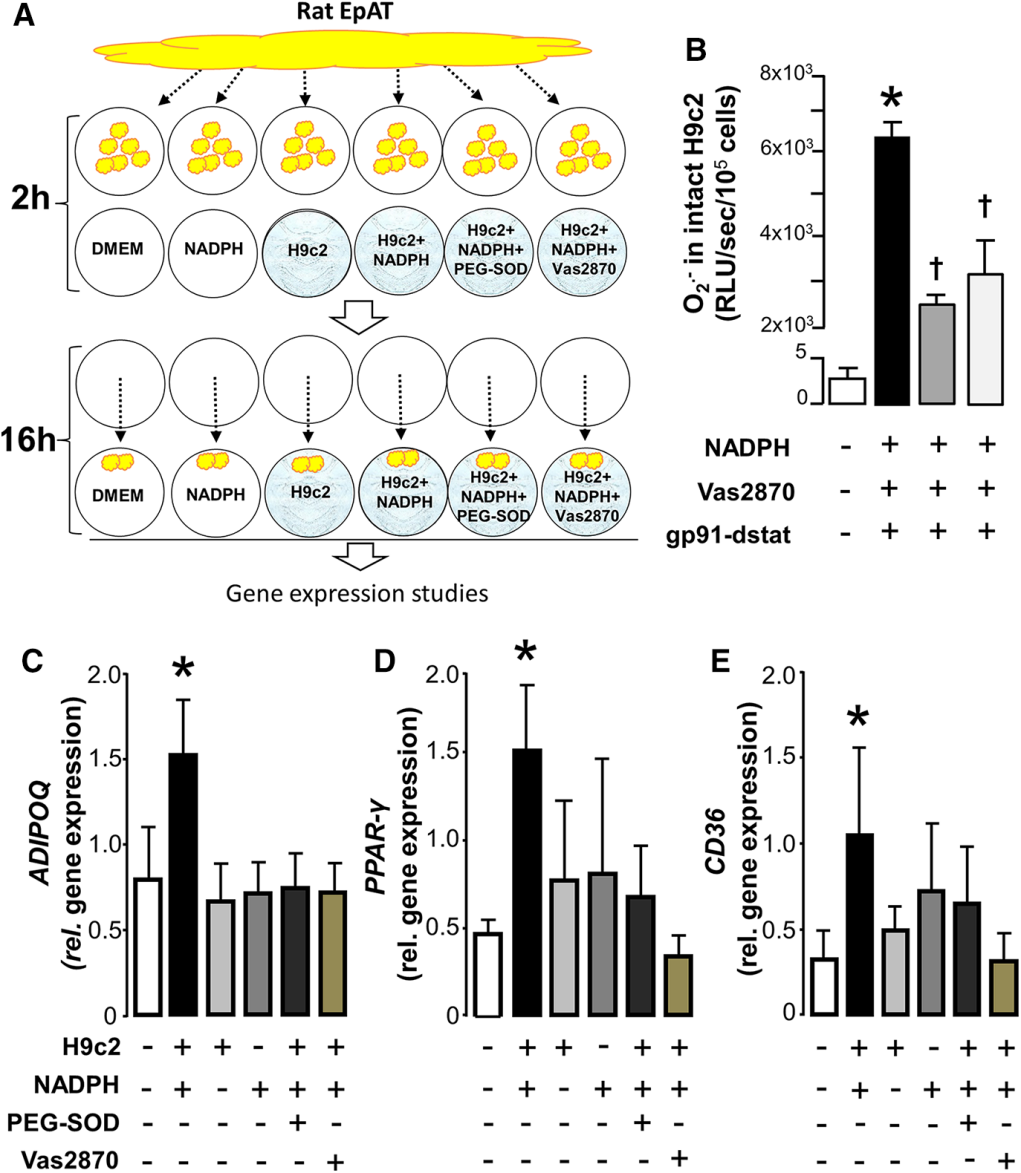
**Activation of nicotinamide adenine dinucleotide phosphate (NADPH) oxidase in H9C2 cardiomyocytes triggers peroxisome proliferator-activated receptor gamma (PPAR-γ) signaling in rat pericardial fat: identifying a novel inside-to-outside signal.** To examine whether under conditions of increased endogenous oxidative stress cardiac myocytes release a transferable factor able to affect the activation of PPAR-γ/adiponectin signaling in rat epicardial adipose tissue (EpAT), we exposed H9c2 cells (differentiated to cardiac myocytes) to NADPH 100 μmol/L for 2 h, whereas rat EpAT was conditioned ex vivo (**A**). After 2 h, the rat EpAT was transferred into the H9c2 wells and cocultured for an additional 16 h (**A**). At the end of the incubation period, gene expression was studied in the rat EpAT. Addition of NADPH to intact H9c2 cells grown on coverslips led to a striking increase of NADPH oxidase–derived superoxide (O_2_^−^) that was partly inhibitable by either Vas2870 (a pan-Nox inhibitor) or gp91-dstat (a specific inhibitor of NOX2; **B**), as demonstrated by real-time monitoring using lucigenin-enhanced chemiluminescence. Coincubation of rat EpAT with H9c2 cardiac myocytes stimulated with NADPH resulted in an upregulation of *ADIPOQ* (**C**), *PPAR-γ* (**D**), and *CD36* (**E**) in EpAT at 16 h. All these effects were prevented by polyethylene glycol (PEG)-SOD (300 U/mL) or vas2870 (10 nmol/L). The presence of unstimulated H9C2 cells or NADPH alone had no effect on the expression of *ADIPOQ, PPAR-γ*, or *CD36* genes in the rat EpAT (**C**–**E**). Concentration of gp91-dstat was 50 μmol/L. Values are presented as mean±SEM. **B**, n=7; (**C**–**E**), n=7; *P<0.05 vs control group; ***P<0.0001 vs resting; †*P*<0.05 vs NADPH alone. DMEM indicates Dulbecco’s Modified Eagle Medium; RLU, relative light units; and SOD, superoxide dismutase.

To explore the hypothesis that the release of a transferable factor from cardiac myocytes under conditions of increased oxidative stress may drive PPAR-γ/*ADIPOQ* expression in the EpAT, we exposed H9c2 cells to NADPH (100 μmol/L, the substrate of NADPH-oxidases) for 2 hours (Figure 4A), as a means to increase the production of O_2_^−^ from NADPH oxidases in these cells (Figure 4B and 4C). Exogenous NADPH increased intracellular NADPH by 25% (Online Figure III). The NADPH-simulated O_2_^−^ in these cells was inhibited by ≈60% using pan-Nox inhibitor Vas2870 and by 50% using the specific Nox2 inhibitor gp91-dstat (Figure 4). At the end of the 2-hour incubation period with NADPH, we cocultured freshly collected rat EpAT with the stimulated H9c2 cells for 16 hours (Figure 4A). At the end of the incubation period, we measured gene expression in the EpAT from coculture. There was no change in the expression of *ADIPOQ*, *PPAR-γ*, or *CD36* genes in rat EpAT incubated with NADPH alone or cocultured with H9c2 cells without added NADPH (Figure 4D–4F). However, coculture of rat EpAT with H9c2 cells prestimulated with NADPH resulted in a significant increase in *ADIPOQ* gene expression (Figure 4D) as well as expression of PPAR-γ (Figure 4E) and its downstream target CD36 (Figure 4F). These effects were all prevented by the O_2_^−^ scavenger PEG-SOD or the NADPH oxidases inhibitor Vas2870 (Figure 4D–4F). These findings indicate that increased O_2_^−^ production from H9c2 cells leads (either directly or indirectly via an intermediate factor) to upregulation of PPAR-γ/adiponectin expression in rat EpAT.

As an alternative model to stimulate O_2_^−^ generation in H9C2 cells in the above experiment, we repeated the same experimental design by using PMA 160 nmol/L instead of NADPH (Online Figure IV). We observed a similar upregulation of *ADIPOQ* and *PPAR-γ* in rat EpAT that was inhibitable by Vas2870. However, the same effect was also observed in the absence of H9C2 cells, suggesting a direct effect of PMA on PPAR-*γ* signaling in the EpAT (Online Figure IV).

### Myocardial Oxidation Products as Candidate Mediators of Inside-To-Outside Cardiac-Adipose Tissue Signaling.

Because O_2_^−^ is a short-lived, highly reactive molecule, we hypothesized that other longer-lived reactive oxygen species rather than O_2_^−^ itself (eg, H_2_O_2_) or even products of lipid oxidation could act as transferable factors released from cardiac myocytes under oxidative stress conditions to exert a paracrine effect on EpAT. Incubation of human EpAT with H_2_O_2_ (100 μmol/L) suppressed *ADIPOQ* gene expression (Online Figure VIA), suggesting that direct exposure of EpAT to H_2_O_2_ cannot explain the observation in the Clinical Associations Studies that high myocardial oxidative stress is linked to high *ADIPOQ* expression in EpAT (Figure 2E).

**Figure 5.**
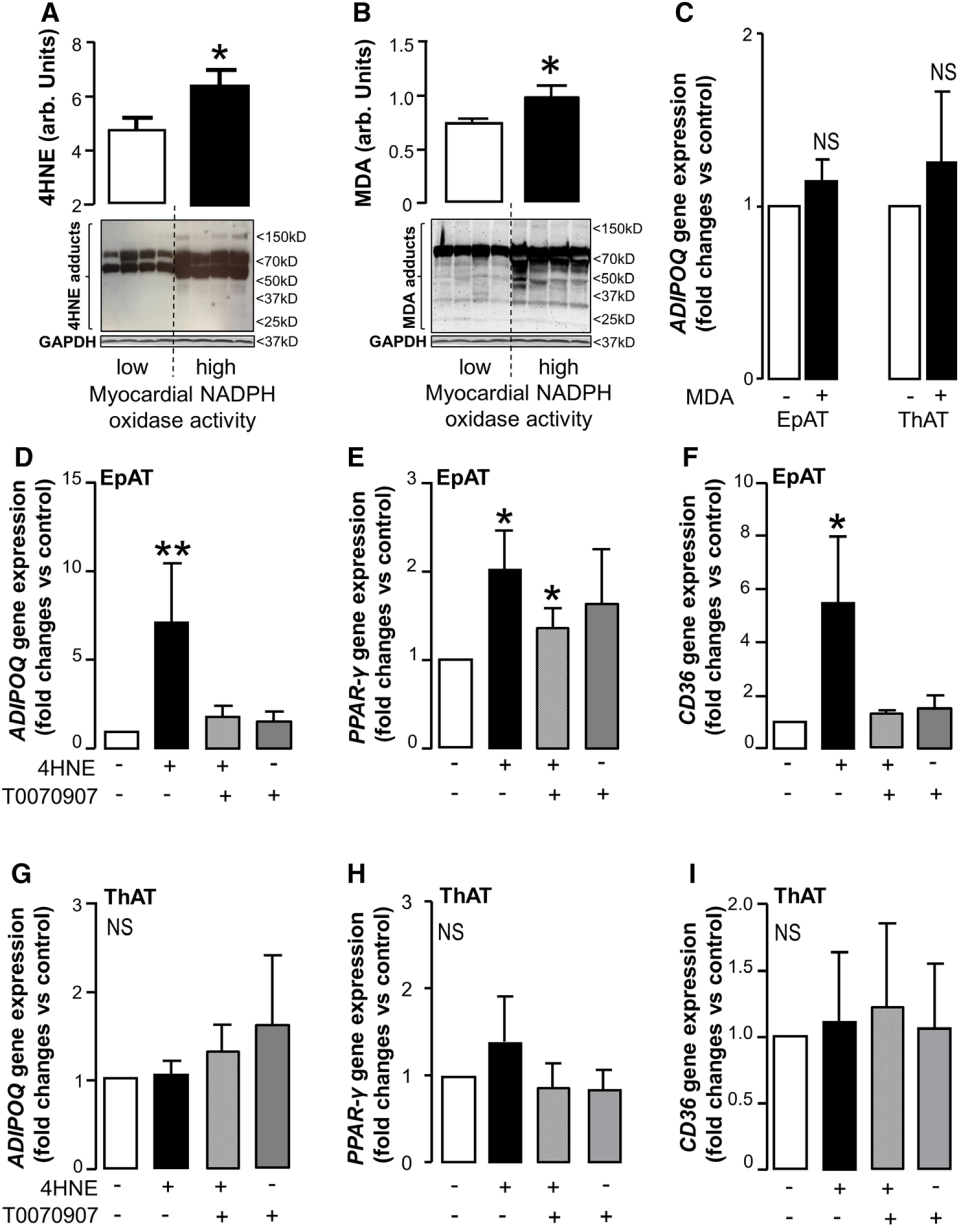
**Myocardial oxidation product 4-hydroxynonenal (4HNE) as a mediator of the inside-to-outside signal from the human myocardium to epicardial adipose tissue (EpAT).** In human myocardium, increased nicotinamide adenine dinucleotide phosphate (NADPH) oxidases activity (upper tertile of NADPH-stimulated O_2_^−^ in the Clinical Associations Studies) were associated with significantly greater 4HNE (**A**) and malonyldialdehyde (MDA; **B**) production. Incubation of EpAT and thoracic (ThAT) adipose tissue with MDA (1 mmol/L) for 16 h had no significant impact on *ADIPOQ* gene expression (**C**). On the contrary, incubation of EpAT with 4HNE (30 µmol/L) for 16 h induced a striking increase in *ADIPOQ* (**D**), *PPAR-γ* (**E**) and *CD36* (**F**). The effects of 4HNE on *ADIPOQ* and *CD36* gene expression were reversed by the inhibitor of PPAR-γ activity, T0070907 (10 μmol/L; **D** and **F**). Importantly, 4HNE had no significant impact on the expression of *ADIPOQ* (**G**), *PPAR-γ* (**H**), or *CD36* (**I**) genes in ThAT. Values are represented as fold change compared to control group (mean±SEM). **A** and **B**, n=4 per group; (**C**) n=5 per group; (**D**–**I**), n=5 to 8 per group, *P<0.05; **P<0.01 vs low myocardial NADPH oxidase activity (**A** and **B**) or control (**C** to **I**).

Next, we examined whether products of lipid oxidation (formed under conditions of high myocardial oxidative stress) could modulate *ADIPOQ* expression in EpAT. We found that increased activity of NADPH oxidases in the human myocardium leads to increased formation of common oxidation products, such as 4HNE and malonyldialdehyde, which form detectable adducts with proteins in the human myocardium (Figure 5A and 5B). 4HNE adducts are also rapidly increased in human myocardial tissue after ex vivo exposure to NADPH (100 μmol/L; Online Figure V). Incubation of human EpAT and ThAT with malonyldialdehyde had no impact on *ADIPOQ* (Figure 5C), *PPAR-γ*, or *CD36* gene expression (Online Figure VII) in either adipose tissue depot. By contrast, exposure of human EpAT to 4HNE induced a rapid 7-fold upregulation of *ADIPOQ* gene expression, an effect that was prevented by the PPAR-*γ* activity inhibitor T0070907 (Figure 5D). 4HNE also induced a respective 2-fold upregulation of *PPAR-γ* gene expression (Figure 5E) and a 5-fold increase of its downstream gene, *CD36* (Figure 5F), the latter being prevented by the use of T0070907 (Figure 5F). Interestingly, the expression of PPAR-γ was significantly higher in EpAT than in ThAT, further supporting the notion that its expression in EpAT is largely driven by local signals released from the human myocardium (Online Figure VIII). Taken together, these findings suggest that 4HNE exerts its effects on *ADIPOQ* gene expression not only by upregulating PPAR-γ but also by enhancing its downstream signaling (Figure 5E and 5F). Interestingly, 4HNE had no effect on the expression of *ADIPOQ*, PPAR-γ, and CD36 genes in remote sites of adipose tissue such as ThAT (Figure 5G–5I), supporting the hypothesis that 4HNE released from the human heart under conditions of increased oxidative stress may activate PPAR-γ signaling in EpAT via a paracrine mechanism.

### Testing the Paracrine Effects of the Heart on EpAT Using Animal Models

To further explore whether a selective increase in myocardial NADPH oxidases activity would lead to upregulation of *ADIPOQ* gene expression in EpAT in vivo, we used a transgenic mouse model with cardiac myocyte–specific overexpression of human *NOX2* (*mNOX2*-Tg; Online Figure IX)^26^ given the strong association of the latter with myocardial redox state in humans (Online Figure X).

**Figure 6.**
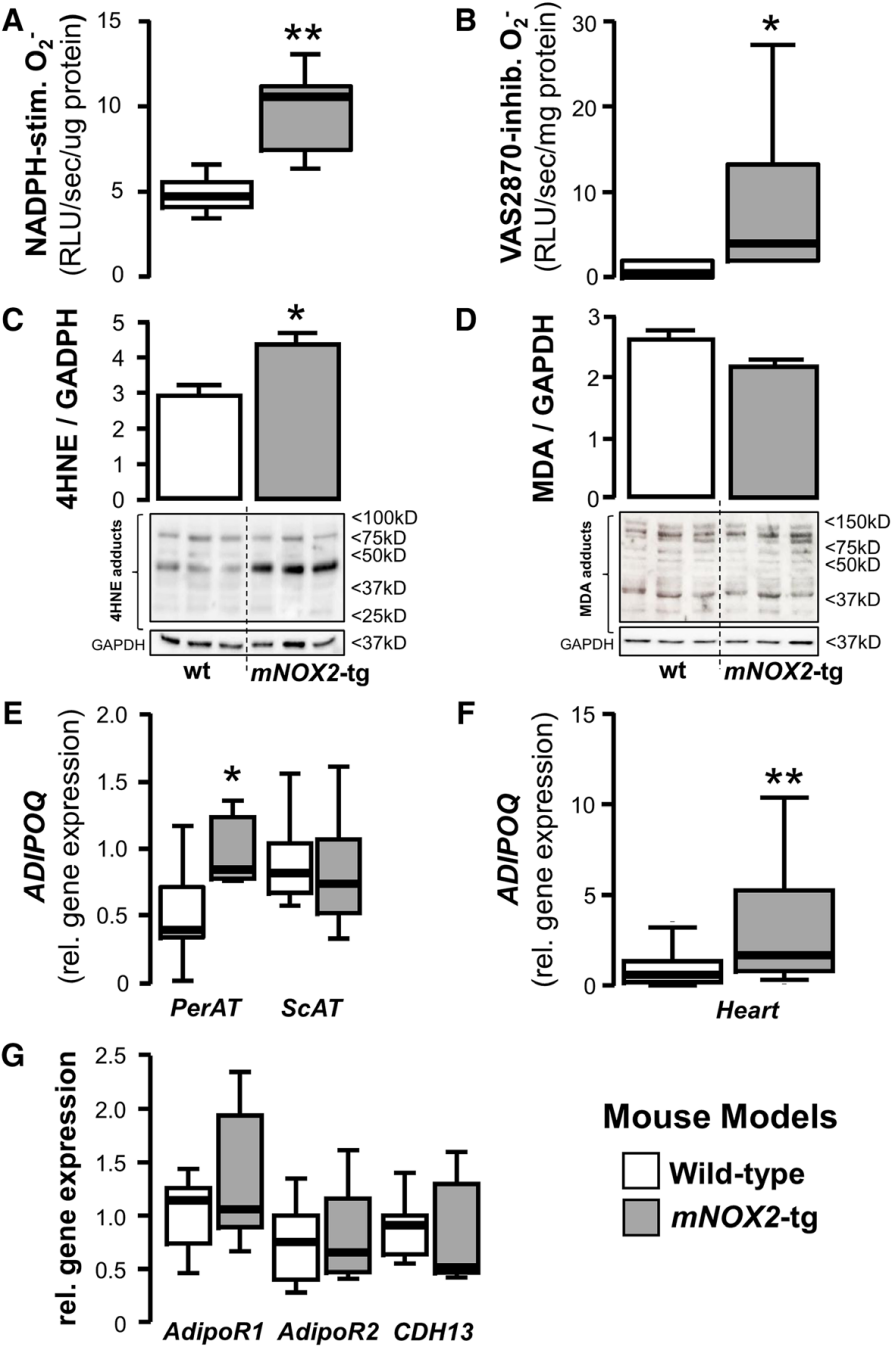
**Testing the inside-to-outside paracrine effects of the heart on pericardial adipose tissue using a cardiomyocyte-specific Nox2-tg mouse model.** In the cardiac myocyte–specific NOX2-transgenic mouse, myocardial nicotinamide adenine dinucleotide phosphate (NADPH) oxidases were activated, as assessed by both the NADPH-stimulated (**A**) and Vas2870-inhibitable superoxide (O_2_^−^) signal (**B**), and by increased formation of 4-hydroxynonenal (4HNE) protein adducts when compared with wild-type (wt) animals (**C**). There was no difference in the myocardial protein levels of malonyldialdehyde (MDA) adducts (**D**). Increased myocardial oxidative stress and 4HNE adducts formation in *mNOX2*-tg mice led to increased *ADIPOQ* gene expression in the fat attached to the heart (pericardial adipose tissue [PerAT]), but not in remote AT depots, eg, subcutaneous AT (ScAT; **E**). *mNOX2*-tg mice also had increased endogenous levels of *ADIPOQ* gene expression in myocardial tissue (**F**), but there was no difference in the myocardial gene expression levels of any of adiponectin receptors, T-cadherin (CDH13), AdipoR1 and AdipoR2 (**G**); (**A**–**E**), n=5 to 6 per group; (**F** and **G**) n=9 to 10 per group, *P<0.05, **P<0.01 vs wt group. RLU indicates relative light units.

Subcutaneous and pericardial AT was collected from 20-week old male *mNOX2*-Tg and wild-type littermate mice. Overexpression of *NOX2* in the myocardium of these mice (demonstrated in Online Figure IX) resulted in a significant increase of NADPH oxidases activity, as assessed by both the NADPH-stimulated O_2_^−^ (Figure 6A) and Vas2870-inhibitable O_2_^−^ production (Figure 6B). Overexpression of *NOX2* also led to increased formation of 4HNE—but not of malonyldialdehyde—adducts in the myocardium of *mNOX2*-Tg mice (Figure 6C and 6D). Interestingly, there was a striking upregulation of *ADIPOQ* expression in pericardial but not in subcutaneous AT of the *mNOX2*-tg mice (Figure 6E). Myocardial *NOX2* overexpression also led to increased *ADIPOQ* gene expression in the myocardium (Figure 6F), but did not affect the expression of adiponectin receptors in the myocardium (Figure 6G).

**Figure 7.**
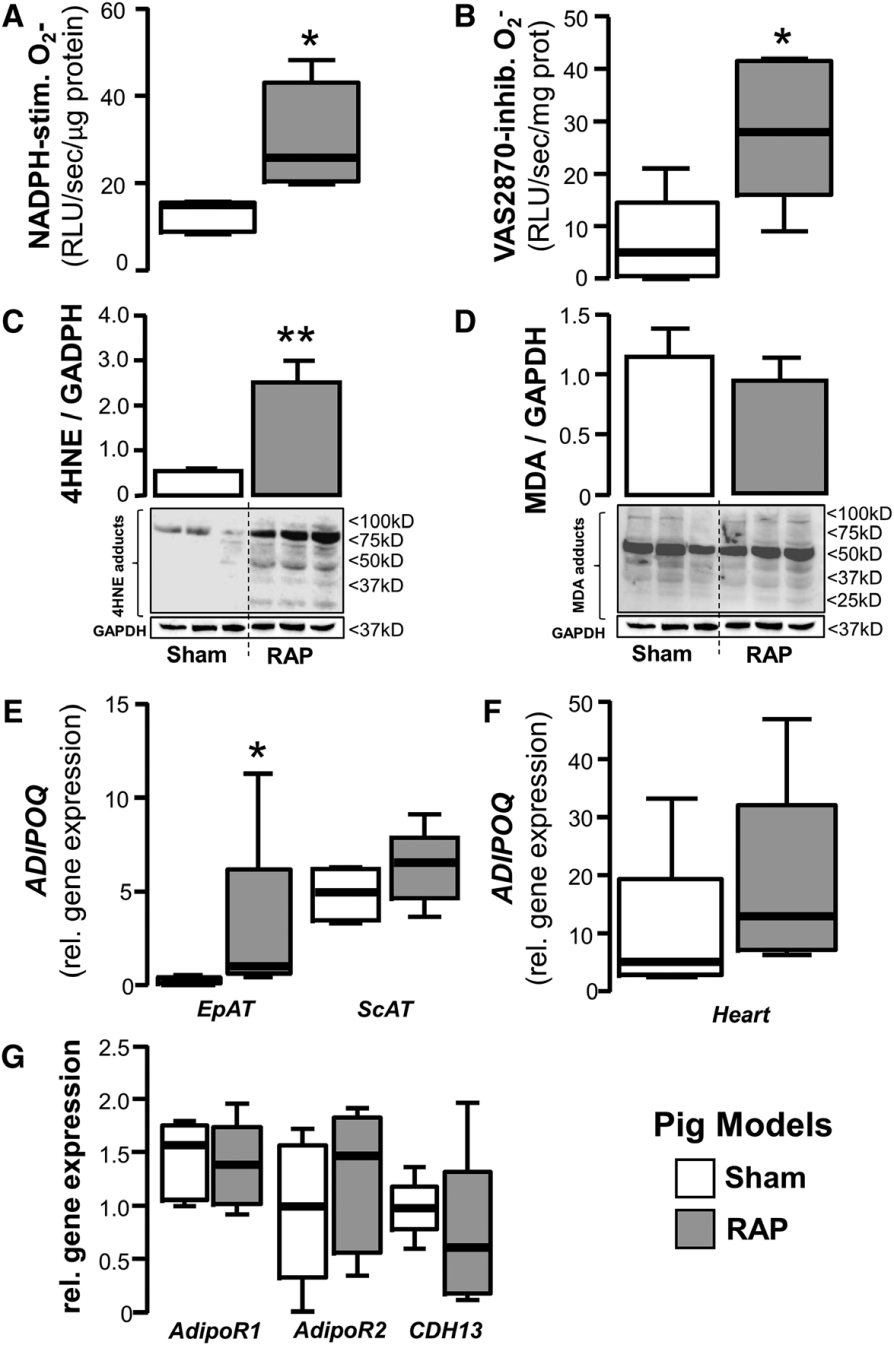
**Testing the inside-to-outside paracrine effects of the heart on adipose tissue using a pig model of rapid atrial pacing (RAP).** In a pig model of RAP, myocardial nicotinamide adenine dinucleotide phosphate (NADPH) oxidases activity was significantly higher in the paced animals than in sham, as shown by both the NADPH-stimulated and Vas2870-inhibitable superoxide (O_2_^−^) signal (**A** and **B**). RAP also increased formation of 4-hydroxynonenal (4HNE; but no malonyldialdehyde [MDA]) protein adducts (**C** and **D**) and upregulated *ADIPOQ* gene expression in epicardial AT (EpAT) but not in remote AT depots, eg, ScAT (**E**). There was no difference in endogenous *ADIPOQ* gene expression levels in the heart (**F**) or myocardial expression of adiponectin receptors (**G**) between sham-operated and RAP animals; all panels, n=5 per group, *P<0.05, **P<0.01 vs sham group. RLU indicates relative light units.

To test whether the findings from the *mNOX2*-Tg mouse model could be replicated in a large animal, with typical EpAT distribution closer to the human one, we used rapid atrial pacing in the pig to increase myocardial O_2_^−^ production. As we have previously shown,^4^ rapid atrial pacing increases myocardial NADPHoxidase activity in the left atrium as assessed by both the NADPH-stimulated O_2_^−^ and the Vas2870-inhibitable O_2_^−^ signal (Figure 7A and 7B). Activation of atrial NADPH oxidase resulted in increased myocardial formation of 4HNE but not of malonyldialdehyde adducts (Figure 7C and 7D, respectively) and led to a striking upregulation of *ADIPOQ* expression in EpAT attached to the left atrium, whereas there was no significant effect on *ADIPOQ* expression in the subcutaneous adipose tissue (Figure 7E), or on the expression of endogenous adiponectin (Figure 7F) or adiponectin receptors (Figure 7G) in the left atrium. Taken together, these data demonstrate that increased NADPH oxidase activity in the heart leads to upregulation of *ADIPOQ* expression specifically in EpAT. This corroborates the findings from our cell culture and ex vivo work in human atrial tissue and explains the positive associations between *ADIPOQ* expression in EpAT and NADPH oxidase activity in the underlying myocardium of patients with IHD.

## Discussion

In the present study, we explore for the first time in humans, the role of adipose tissue-derived adiponectin in the regulation of myocardial redox state, through modulation of NADPH oxidase activity. Adiponectin prevents the phosphorylation and membrane translocation of p47^phox^ and prevents Rac1 activation and membrane translocation, both key aspects of myocardial NADPH oxidase activation. Under conditions of increased myocardial O_2_^−^ generation, oxidation products such as 4HNE can act as signaling molecules from the heart, to activate adiponectin release from EpAT in a PPAR-γ–dependent manner. This introduces the novel concept that, through the release of oxidation products, the human heart regulates key biological processes in adipose tissue by triggering rescue PPAR-γ signaling leading to increased release of adiponectin, which then exerts cardioprotective effects. This novel inside-to-outside signal may be a therapeutic target for the prevention and treatment of redox-dependent cardiac diseases.

Myocardial redox state is a critical determinant of cardiac biology by modulating the function of ion channels, sarcoplasmic reticulum calcium release channels, and myofilament proteins, in cardiac myocytes.^1,2,27^ O_2_^−^ causes damage to cell membranes and leads to cardiac myocyte necrosis and apoptosis,^2,28^ whereas redox-sensitive signaling pathways control fibrotic and hypertrophic responses.^29^ NADPH oxidases and particularly Nox2 are major contributors to O_2_^−^ production in the cardiovascular system,^30^ leading to the development of multiple cardiac pathologies such as heart failure,^29^ myocardial hypertrophy,^6^ atrial fibrillation,^3–5^ and others. Nox2 activation is dependent on Rac1 binding with GTP as well as p47^phox^ phosphorylation and their subsequent translocation to the membrane to form the active catalytic complex of the enzyme.^31^ In this study, we demonstrate that systemic oxidative stress (as characterized by plasma malonyldialdehyde) is not significantly correlated with NADPH oxidase activity in human myocardium, confirming our previous observation that myocardial redox state is independent of markers of systemic oxidative stress and may be subjected to local, largely unknown regulatory mechanisms.^32^

Human adipose tissue secretes a wide range of adipocytokines able to affect myocardial biology.^16,33^ In addition, EpAT has been proposed to exert paracrine effects on the underlying epicardial coronaries.^34^ Given the close anatomic relationship between myocardium and EpAT in humans (with adipose tissue penetrating into the heart muscle), it seems that EpAT exerts paracrine effects on the underlying myocardium affecting its biology, as recently demonstrated in some elegant translational studies by Greulich et al.^33^ Adiponectin seems to have some direct effects on myocardial redox state in animal and cell culture models,^28,35^ but its role in the regulation of redox state in the human heart is unknown. It is also unclear whether adiponectin produced in EpAT has any paracrine role in the regulation of myocardial redox state, as suggested above.

In this study, we first evaluated the association between myocardial NADPH oxidases activity and circulating adiponectin or the expression of adiponectin from EpAT or ThAT in a cohort of patients with IHD. Paradoxically, we observed a positive association between myocardial NADPH-stimulated O_2_^−^ and circulating adiponectin as well as *ADIPOQ* gene expression in EpAT. A similar positive association was also observed between myocardial NADPH-stimulated O_2_^−^ and adiponectin receptor AdipoR1. As this was the first study exploring the role of adiponectin in the regulation of myocardial redox state in humans, we then tried to explore the direction of that unexpected association. By taking advantage of the genetic variability of *ADIPOQ* gene described in recent genome-wide association studies,^24,36^ we observed that genetically determined reduction of adiponectin levels is also associated with increased NADPH-stimulated O_2_^−^ in the heart of patients with IHD (an association independent of other covariates, including BNP), implying that low adiponectin production in the human AT is causally associated with higher myocardial NADPH oxidase activity. Using an ex vivo model of human myocardium, we observed that adiponectin had a direct inhibitory effect on myocardial NADPH oxidase activity (evidenced by a reduction in Vas2870-inhibitable myocardial O_2_^−^) and that this effect is mediated by the activation of AMPK leading to reduced phosphorylation and membrane translocation of NADPH oxidase subunit p47^phox^ in parallel to an AMPK-mediated suppression of Rac1 activation and membrane translocation. This is the first study defining the molecular mechanisms by which adiponectin suppresses NADPH oxidase activity in the human heart, but these findings are still unable to explain the positive association observed between myocardial redox state and *ADIPOQ* gene expression in EpAT in patients with IHD.

In advanced cardiovascular disease states (ie, heart failure), circulating adiponectin is significantly elevated.^37^ In cell culture studies,^36^ it was demonstrated that adipocytes respond to exogenous BNP (which is significantly elevated in heart failure) by upregulating *ADIPOQ* gene expression, and we have recently demonstrated that this stimulatory effect of BNP on human adipose tissue over-rides the suppressive effect of inflammation on the expression and release of adiponectin.^14^ Although BNP seems to drive the circulating adiponectin level in the presence of heart failure, its role in the regulation of adipose tissue biology in the absence of heart failure seems less important.^14^ In this study, we demonstrate that both circulating adiponectin and *ADIPOQ* gene expression in EpAT are positively correlated with myocardial redox state (and specifically NADPH oxidase activity), independently of plasma BNP. Importantly, we demonstrate for the first time that products of oxidation, such as 4HNE, which is produced in the human heart under conditions of increased oxidative stress and can cross cellular membranes and act as signaling molecules,^38^ upregulate *ADIPOQ* gene expression in the human EpAT via a PPAR-γ–dependent mechanism. This novel concept was demonstrated by coculturing human EpAT with differentiated H9c2 cells after stimulation of cellular O_2_^−^ production by supplying the NADPH oxidase with its substrate, ie, NADPH. This finding was then confirmed using a cardiac myocyte–specific *NOX2* transgenic mouse and a pig model of rapid atrial pacing (as means to increase NADPH oxidases activity and O_2_^−^ generation in the atrial myocardium). Interestingly, in all models, it was consistently demonstrated that under conditions of increased myocardial O_2_^−^ generation, the myocardium releases transferable factor(s) (one of which seems to be 4HNE), which then activates PPAR-γ signaling and upregulates adiponectin expression specifically in the neighboring EpAT but not in other remote adipose tissue depots such as ThAT. Upregulation of adiponectin in EpAT represents a protective paracrine response of EpAT against myocardial oxidative stress, by inhibiting myocardial NADPH oxidase. With these findings, we document that EpAT hosts local defense mechanisms protecting the heart against oxidative stress (Online Figure XI).

Our study has some limitations. The patients included into the Clinical Associations and the ex vivo study arms were matched for age and sex, but there are some differences in other demographics and risk factor profile. Although we do not perform any direct comparisons between the 2 study arms, any extrapolations of the results from the ex vivo study arm to the Clinical Associations Studies should be made with caution. Moreover, the use of CC for pharmacological inhibition of AMPK has been criticized because of possible non-AMPK–specific effects^39^; nevertheless, it remains the most widely used cell-permeable AMPK inhibitor in cell/tissue experiments. We hypothesized that the lack of any effects of increased myocardial O_2_^−^ generation/4HNE release on subcutaneous adipose tissue is because of its remote anatomic site, but it could be also related to local depot-specific mechanisms maintaining increased *ADIPOQ* expression in this, preventing any further upregulation of *ADIPOQ* by exogenous 4HNE.^14^ Finally, although we have shown that exogenous NADPH increases intracellular NADPH and stimulates O_2_^−^ generation from NADPH oxidases, these enzymes have their NADPH-binding site intracellularly,^40–42^ and as NADPH is a large and charged molecule, it is unlikely to cross the plasma membranes by simple diffusion. Therefore, the exact mechanism by which exogenous NADPH triggers O_2_^−^ generation from NADPH oxidases in H9c2 cells used in the coculture experiment with rat epicardial fat is unclear. The use of the PMA as a stimulus for O_2_^−^ generation in H9C2 cells (which is know to stimulate NADPH oxidases in a protein kinase C-α–mediated mechanism) has also direct effects on PPAR-γ signaling in the rat EpAT; therefore, the result from that experiment should be interpreted with caution.

In conclusion, we demonstrate for the first time that adiponectin inhibits NADPH oxidase in the human myocardium via an AMPK/Rac1/p47^phox^-mediated signaling. We also show that under conditions of increased myocardial oxidative stress, the heart releases transferable mediators (eg, products of oxidation such as 4HNE), which may diffuse to EpAT leading to PPAR-γ–dependent upregulation of adiponectin expression in EpAT. This feedback loop represents a novel defense mechanism of the human heart against myocardial oxidative stress and may prove to be a rational therapeutic target in cardiac disease.

## Sources of Funding

This work was supported by a Marie Curie Intra European Fellowship by European Commission within the 7th Framework Programme Research (Acronym Project HRS-EAT, 300289) and by grants from the British Heart Foundation Centre of Research Excellence-Oxford (RE/08/004), the British Heart Foundation (FS/11/66/28855, PG/13/56/30383, and RG/11/15/29375), the King’s College London British Heart Foundation Centre of Research Excellence (RE/13/2/30182) and by the National Institute for Health Research Oxford Biomedical Research Centre.

## Disclosures

None.

## Supplementary Material

## Novelty and Significance

### What Is Known?

- Nicotinamide adenine dinucleotide phosphate oxidases are major contributors to myocardial oxidative stress in humans.
- Epicardial adipose tissue is an endocrine tissue that secretes active adipokines.
- In experimental animal models, adipose tissue–derived adiponectin has beneficial effects on cardiovascular system.
