# Supplementary material for: Mutual Regulation of Epicardial Adipose Tissue and Myocardial Redox State by PPAR-γ/Adiponectin Signalling
Source: Circ Res. 2016 Mar 3;118(5):842–55. doi: 10.1161/CIRCRESAHA.115.307856 (PMC4772814; doi:10.1161/CIRCRESAHA.115.307856)
Supplement: Supplementary file 1 [file res-118-842-s001.pdf]

SUPPLEMENTAL MATERIAL

Mutual Regulation of Epicardial Adipose Tissue and Myocardial Redox State by PPAR-γ /Adiponectin Signalling

Alexios S. Antonopoulos[a] , Marios Margaritis[a], Sander Verheule[b], Alice Recalde[a], Fabio Sanna[a], Laura Herdman[a], Costas Psarros[a], Hussein Nasrallah[b], Patricia Coutinho[a], Ioannis Akoumianakis[a], Alison C. Brewer[d], Rana Sayeed[e], George Krasopoulos[e], Mario Petrou[e], Akansha Tarun[a], Dimitris Tousoulis[c], Ajay M. Shah[d], Barbara Casadei[a], Keith M. Channon[a], Charalambos Antoniades[a]

[a]Division of Cardiovascular Medicine, Radcliffe Department of Medicine, University of Oxford, United Kingdom
[b]Cardiac Electrophysiology Group, Department of Physiology, Maastricht University, Netherlands
[c]Department of Cardiology, Athens University Medical School, Athens, Greece
[d]Cardiovascular Division, King's College London BHF Centre, London, United Kingdom
[e]Department of Cardiac Surgery, John Radcliffe Hospital, Oxford United Kingdom

1st Author's Surname: Antonopoulos
Short title: Epicardial adipose tissue and myocardial redox

Corresponding author
Charalambos Antoniades MD PhD
Associate Professor of Cardiovascular Medicine
Division of Cardiovascular Medicine, University of Oxford
John Radcliffe Hospital, Oxford OX3 9DU, United Kingdom
Tel: +44-1865-228340, Fax: +44-1865-740352
e-mail: antoniad@well.ox.ac.uk

Detailed Methods

Blood Sampling and Circulating Biomarkers Measurements

Venous blood samples were obtained after 8 hours of fasting, at the morning of the operation for measurements of circulating biomarkers. After centrifugation at 2000 g / 4°C for 15 min, plasma or serum was collected and stored at −80 °C until assayed. Whole blood was also collected for genotyping. Serum adiponectin and interleukin 6 (IL-6), a marker of inflammation, were measured using enzyme linked immunosorbent assays (ELISA) (BioVendor, Brno, Czech Republic and R&D systems USA respectively). Plasma malonyldialdehyde (MDA), a marker of systemic oxidative stress, was quantified by using the TBARS fluorometric assay, as previously described.[1] High sensitivity C-reactive protein (hsCRP), another marker of inflammation, was measured by the high-sensitivity latex enhanced immunoturbidimetric assay (ADVIA, Bayer HealthCare LLC). Plasma BNP was quantified by chemiluminescent-microparticle immunoassay (Architect BNP, Abbott, Germany).

DNA Extraction and Genotyping

Genomic DNA was extracted from whole blood using standard methods (QIAamp DNA blood Midi kit, Qiagen). Genotyping for the rs17366568 (functional polymorphism in *ADIPOQ* gene) and rs266717 SNPs (functional polymorphism in *ADIPOQ* gene promoter) was performed using TaqMan probes (Applied Biosystems; Assay IDs: C-33187752-10 and C-8288442-10 respectively). The assay was run on an ABI StepOne Plus PCR system according to the manufacturer’s protocol.

Harvesting of Human Myocardium and Adipose Tissue Samples

During CABG, myocardial tissue samples were collected from the site of right atrial appendage (RAA) as we have previously described, and transferred into oxygenated (95% O_2 / 5% CO_2) ice-cold buffer. Samples of EpAT were harvested from the site of the right atrioventricular groove (inside the pericardial sac, attached to the heart), while thoracic adipose tissue (ThAT) samples were harvested from outside the pericardium (as “control” samples to the EpAT) and transferred in ice-cold phosphate buffer saline. All tissue samples were transferred immediately to the lab and either used for *ex-vivo* experiments or stored at -80°C for other studies as described below.

Myocardial Superoxide Measurements

Myocardial $O_2^{\cdot-}$ production was measured in samples of right atrium appendages using lucigenin (5μmol/L)-enhanced chemiluminescence, as we have previously described.[1] Myocardial tissue was homogenised in ice-cold Krebs HEPES Buffer pH 7.35 in the presence of protease inhibitor (Roche Applied Science, Indianapolis, IN) using a pre-cooled Polytron homogeniser. The contribution of NADPH oxidase activity to myocardial $O_2^{\cdot-}$ production was quantified in the presence of NADPH 100μmol/L. The use of homogenates allows us to overcome issues regarding penetration of NADPH (which is a polar molecule) into the cells or tissue. In certain *ex vivo* experiments with myocardial tissue, Vas2870 (40 μmol/L, a specific pan NADPH oxidase inhibitor[2]) was also used to get the Vas2870-inhibitable $O_2^{\cdot-}$ signal as a more specific index of NADPH oxidase activity. In a pilot experiment comprising 10 RAA samples, we found that VAS2870 inhibitable $O_2^{\cdot-}$ (pan-Nox inhibitor) was co-linear to the gp91dstat-inhibitable $O_2^{\cdot-}$ (specific for Nox2) with r=0.8875 and P=0.0012. Therefore the use of vas2870 as an NADPH-oxidase inhibitor in the human right atrial appendage provides information mainly on Nox2-derived $O_2^{\cdot-}$.

DHE Staining Method

In situ $O_2^{\cdot-}$ production was determined in right atrial appendage cryosections with the oxidative fluorescent dye dihydroethidium (DHE) as previously described.[3, 4] Briefly, myocardial tissue was washed in ice-cold Krebs HEPES buffer and then cut into thin strips containing all myocardial layers. The tissue was first equilibrated for 20min in Krebs HEPES Buffer pH 7.35 at 37°C and then incubated for 2 hours in the presence or absence of recombinant full-length adiponectin 0.3 μmol/L (10 μg/ml, BioVendor) +/- CC (10 μmol/L). At the end of the incubation period tissue was collected and snap frozen in OCT. Myocardial cryosections (30μm) were equilibrated in Krebs Hepes buffer with or without Vas2870 (40μmol/L) or peg-SOD (300 U/ml). Then the samples were incubated with

DHE (2μmol/L for 5 minutes). Fluorescence images of the myocardium (x63, Zeiss LSM 510 META laser scanning confocal microscope) were obtained from each myocardial tissue quadrant. In each case, segments of myocardial tissue (with and without Vas2870 or peg-SOD) were analyzed in parallel with identical imaging parameters. DHE fluorescence was quantified by using Image-Pro Plus software (Media Cybernetics), while all analyses were performed in a blinded fashion.

RNA Isolation and Quantitative Real Time-Polymerase Chain Reaction (qRT-PCR) Samples of adipose tissue and myocardial tissue samples were snap frozen in QIAzol (Qiagen, Stanford, CA) and stored at -80°C until processed. RNA was extracted by using the RNeasy Micro or Mini kit (Qiagen). RNA was converted into complementary DNA (Quantitect Rev. Transcription kit - Qiagen), then subjected to qPCR using TaqMan probes (Applied Biosystems, Foster City, CA). The reactions were performed in triplicate in 384-well plates, using 5 ng of cDNA per reaction, on an ABI 7900HT Fast Real-Time PCR System (Applied Biosystems). The efficiency of the reaction in each plate was determined based on the slope of the standard curve; relative expression of *ADIPOQ* gene was calculated using the Pfaffl method [5].
For the clinical studies, *PPIA* (cyclophilin) and *PGK1* were used as housekeeping genes for adipose tissue and myocardial tissue, respectively. The Assay IDs of the Taqman probes used were *ADIPOQ* (adiponectin gene): Hs00605917_m1; *ADIPOR1*: Hs01114951_m1, *ADIPOR2*: Hs00226105_m1, *CDH13*: Hs01004530_m1, *CD36*: Hs01567185_m1, *PPARG*: Hs01115513_m1, *PGK1*: Hs00943178_g1, *PPIA*: Hs04194521_s1, *CYBB*(*NOX2*), *NOX4*, *CYBA* (*P22PHOX*), *NCF1 (P47PHOX), NCF2(P67PHOX).*
For the rat epicardial adipose tissue (see below), *PPIA* was used as housekeeping gene. The Assay IDs of the Taqman probes used were *PPIA*: Rn00690933_m1, *ADIPOQ*: Rn00595250_m1, *PPARG*: Rn00440945_m1, *CD36*: Rn00560963_s1.
For the mouse model (see below), *PPIA* and actin-alpha (*ACTA*) were used as housekeeping genes for adipose tissue and myocardial tissue, respectively. The Assay IDs of the Taqman probes used were *ACTA*: Mm00808218_g1, *ADIPOQ*: Mm00456425_m1, *ADIPOR1*: Mm01291334_mH, *ADIPOR2*: Mm01184032_m1, *CDH13*: Mm00490584_m1, *PPIA*: Mm02342429_g1.
For the pig model, *PPIA* and actin-beta (*ACTB*) were used as housekeeping genes for adipose tissue and myocardial tissue, respectively. The assay IDs of the Taqman probes used were *ACTB*: Ss03376160_u1, *ADIPOQ*: Ss03384375_u1, *ADIPOR1*: Ss03378803_u1, *ADIPOR2*: Ss03391825_g1, *CDH13*: Ss03386756_u1, *PPIA*: Ss03394782_g1.

Western Blots in human myocardial samples
To investigate adiponectin-AMPK signaling axis and its effects on NADPH oxidase activity, western immunoblotting was used to examine the direct effects of adiponectin on phospho(Th172)-AMPKa, total AMPKa, phospho(Ser473)-Akt, pan-Akt, phospho(Ser79)-acetyl-CoA carboxylase (ACC) and total ACC (antibodies by Cell Signaling, Danvers, MA), Nox1, Nox2 (antibodies by BD Transduction Laboratories), Nox4, Nox5 (antibodies by Abcam, Cambridge, UK), phospho(Ser359)-p47phox, total p47phox, p67phox (antibodies by Cell Signaling), and total Rac1 (antibody by Merck Millipore, Billerica, MA) protein levels in human myocardium incubated *ex-vivo*. Selected myocardial tissue samples from Clinical Associations Studies were also used to evaluate the content of 4-hydroxynonenal (4HNE) and malonyldialdehyde (MDA) adducts, the two most common lipid oxidation products (formed when reactive oxygen species react with lipid membranes, antibodies by Abcam) in the presence of high or low myocardial NADPH-stimulated $O_2^{.-}$ generation. Briefly, myocardial tissue samples were homogenized for 30 seconds using a pre-cooled electric Polytron homogenizer in 300 μl of lysis buffer (Invitrogen, UK) containing a protease and phosphatase inhibitor cocktail (Roche Applied Science). Homogenates were spun at 13,000 rpm for 10 minutes, at 4 ºC. The protein concentration of the supernatants was then measured using the BCATM Protein Assay kit (Pierce, UK). Protein lysates were separated on 4-12% gradient SDS-NuPAGE gel (Invitrogen, UK), and proteins transferred to polyvinylidene difluoride membranes (Amersham, UK Ltd.), followed by blocking with 5% powdered skimmed milk. The membranes were incubated with the respective primary antibodies overnight and immunodetection of the primary antibodies was performed with horseradish-peroxidase-conjugated secondary antibodies (Promega) and enhanced

chemifluorescence (Amersham Bioscience UK Ltd.) and quantified in relation to the house-keeping protein, GAPDH (Santa Cruz Biotechnology, Santa Cruz, CA).

Measurement of Myocardial Rac1 Activation and Membrane Translocation Experiments

Rac1 activation was evaluated by a commercially available affinity precipitation assay using the PAK1-PBD conjugated glutathione agarose beads (Millipore, Temecula, USA)[3]. To estimate membrane translocation of Rac1 and p47phox, we performed differential centrifugation for isolation of membrane proteins, and membrane-translocated Rac-1 and p47phox proteins were determined by Western immunoblotting as previously described.[3]

Measurement of intracellular NADP/NADPH levels

For measurements of intracellular NADP/NADPH levels, a commercially available fluorometric assay was used (Abcam kit, Cambridge, UK). Following the 18 hour treatment, H9C2 cells were washed 3x with PBS and then lysed. Twenty five μl of the lysates were loaded into a black walled 96-well plate for processing. Finally, 75ul of NADPH reaction mixture was added to initiate the reaction according to manufacturer's instructions and after 45 minutes the fluorescence was measured at Ex/Em=540/590nm. The assay kit provides measurements of intracellular NADPH, NADP and total NADPH/NADP separately.

Animal studies

Generating the mouse model: The cardiomyocyte-specific *NOX2* overexpressing mouse model (*mNOX2*-tg) was generated in the laboratory of Ajay Shah.[6] The expression of the human 1.8kb *NOX2* cDNA was driven by the mouse myosin light chain-2 (MLC-2v) promoter. Transgenic founders were backcrossed for >10 generations onto a C57BL/6 background.

The decision to overexpress human *NOX2* in these mice was based on our findings in Clinical Associations Studies in which *NOX2* gene expression was strongly correlated with myocardial $O_2^{\cdot -}$ in the human right atrium appendages (Online Figure X), while its activation dependents on $p47^{phox}$ phosphorylation/membrane translocation and Rac1 activation. On the other hand, unlike Nox2, Nox4 does not require Rac1/p47phox membrane translocation to be activated, and exploring its role in this setting was beyond the scope of the current study.

Pig model: In 10 Dutch Landrace pigs (62±3 kg), anesthesia was induced with Zoletil (5-8mg/kg i.m.) and Thiopenthal (5-15mg/kg i.v.). After intubation, anesthesia was maintained by intravenous infusion of Midazolam (1.0mg/kg/h), Sufentanyl (4mg/kg/h) and Propofol (2.5-10mg/kg/h). An endocardial lead (Capsurefix 5568, Medtronic, Minneapolis, MN) was implanted in the right atrium and connected to a subcutaneous pacemaker (Itrel II, Medtronic, Minneapolis, MN). Healthy pigs (61±2 kg) served as a control group. After one week recovery, the pacemaker was switched on at a rate of 10Hz for 5 weeks. The ventricular rate was controlled by digoxin 10μg/kg for 1 week, followed by 5μg/kg for 4 weeks. Digoxin was discontinued 3 days before the sacrifice experiment in order to reach plasma levels <0.5μg/ml. At sacrifice, animals were anesthetized as described above and the heart was excised via a left lateral incision. Epicardial adipose tissue was collected from the posterior left atrium, close to the AV ring. For comparison, subcutaneous fat was taken from the incision in the thorax.

Power calculations

Sample size calculations were based on previous data from our laboratory. For the clinical studies, we estimated that a total number of 200 subjects would allow us to detect a 0.31 (or 7.5%) difference in log(myocardial NADPH-stimulated $O_2^{\cdot -}$) between the extreme tertiles of plasma adiponectin with an α=0.05, a power of 0.9, and an assumed standard deviation of 0.57. For the ex vivo experiments, sample size calculations were performed on the basis of pilot experiments and we estimated that with a minimum of 5 pairs of samples (serial samples from the same myocardial tissue), we would be able to identify a change in log(myocardial NADPH-stimulated $O_2^{\cdot -}$) of 0.84 (or 20%) with an α=0.05, a power 0.9, and a standard deviation for a difference in the response of the pairs of 0.44. Power calculations for the animal experiments were based on pilot data on adiponectin expression from epicardial adipose tissue; for the transgenic mice experiments, we estimated that with a minimum of 5 mice per group, we would be able to identify a change in adiponectin gene expression of 0.51 (2fold

change) with an α=0.05, a power 0.9, and a standard deviation for a difference in the response of the pairs of 0.22. Similarly, for the pig model of atrial pacing, we estimated that with a minimum of 5 pigs per group, we would be able to identify a change in adiponectin gene expression from epicardial adipose tissue of 0.91 (3fold change) with an α=0.05, a power 0.9, and a standard deviation for a difference in the response of the pairs of 0.39.

Supplemental Tables

Online Table I: Multivariable models of myocardial NADPH-stimulated $O_2^{\cdot-}$ and serum adiponectin

**Model for myocardial NADPH-stimulated $O_2^{\cdot-}$**		
**Univariate analysis**		
Variable	***Correlation coefficient****	***P-value***
Log(serum adiponectin)	0.361	0.0001
Log(plasma BNP)	-0.045	0.601
Age	0.179	0.031
Male gender	-0.220	0.007
Smoking status	0.153	0.064
Left ventricular ejection fraction	-0.213	0.023
**Multivariable analysis** (R^2 for the model: 0.211, P=0.001)		
Variable	***Standardized beta***	***P-value***
Log(serum adiponectin)	3.802	0.001
Log(plasma BNP)	-0.08	0.465
Age	0.063	0.526
Male gender	-0.086	0.419
Smoking status	0.034	0.734
Left ventricular ejection fraction	-0.163	0.137

**Model for circulating adiponectin**		
**Univariate analysis**		
Variable	***Correlation coefficient****	***P-value***
Log(NADPH stimulated $O_2^{\cdot-}$)	0.361	0.0001
Log(plasma BNP)	0.257	0.0001
Male gender	-0.175	0.008
Age	0.224	0.001
Body mass index	-0.126	0.07
Diabetes mellitus	-0.128	0.05
Hypercholesterolaemia	-0.175	0.009
**Multivariable analysis** (R^2 for the model: 0.215, P=0.0001)		
Variable	***Standardized beta***	***P-value***
Log(NADPH stimulated $O_2^{\cdot-}$)	0.210	0.016
Log(plasma BNP)	0.229	0.008
Male gender	-0.210	0.017
Age	-0.042	0.082
Body mass index	-0.148	0.225
Diabetes mellitus	-0.067	0.423
Hypercholesterolaemia	-0.102	0.628

BNP: Brain natriuretic peptide; *Spearman’s or Pearson’s correlation coefficient as appropriate

Supplemental Figures and Figure Legends

Online Figure I

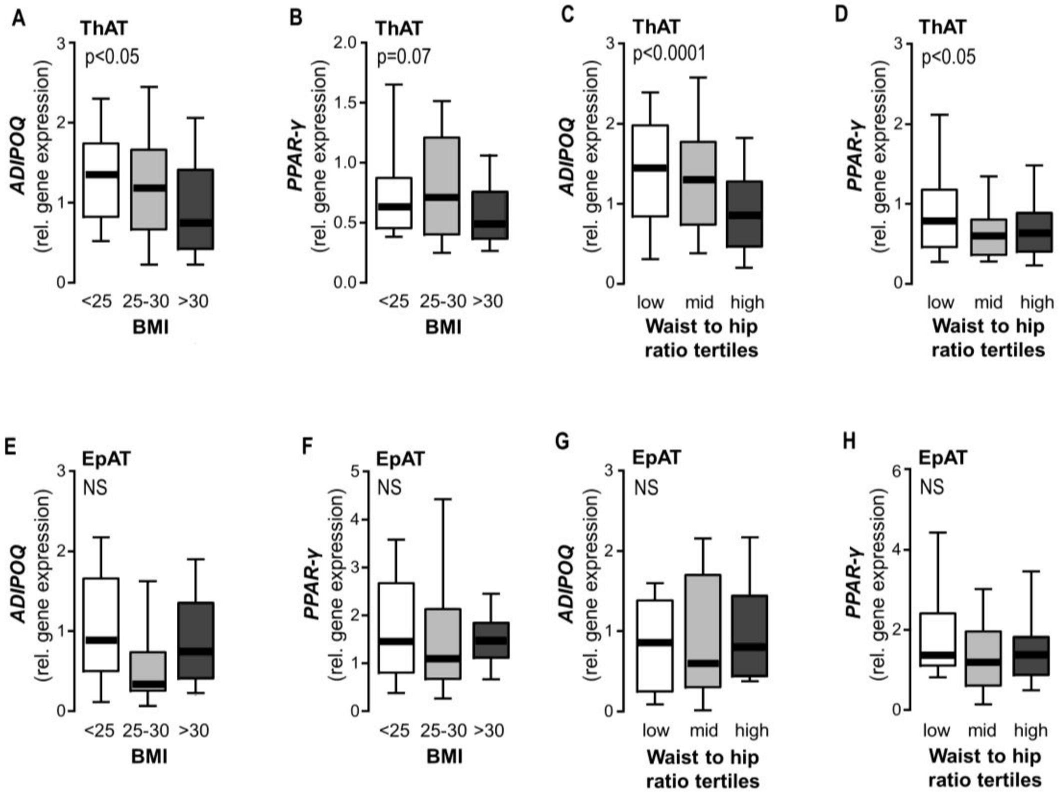

Obesity and adiponectin/PPAR-γ gene expression in thoracic and epicardial adipose tissue. In patients of Clinical Associations Studies (clinical cohort of 247 patients undergoing coronary artery bypass grafting), there was a significant inverse association between increased body mass index (BMI) and adiponectin gene (*ADIPOQ*) expression from thoracic adipose tissue (AT, Panel A). A trend towards lower PPAR-γ gene expression from thoracic AT with increased BMI was observed too but this did not reach statistical significance (Panel B). Abdominal obesity as assessed by the waist to hip ratio was also associated with lower *ADIPOQ* (Panel C) and *PPAR-γ* gene expression (Panel D) from thoracic AT. On the other hand, BMI and waist to hip ratio were not associated with adiponectin (Panel E & G) or *PPAR-γ* gene expression (Panel F & H) in epicardial AT. p-values are derived from ANOVA.

Online Figure II

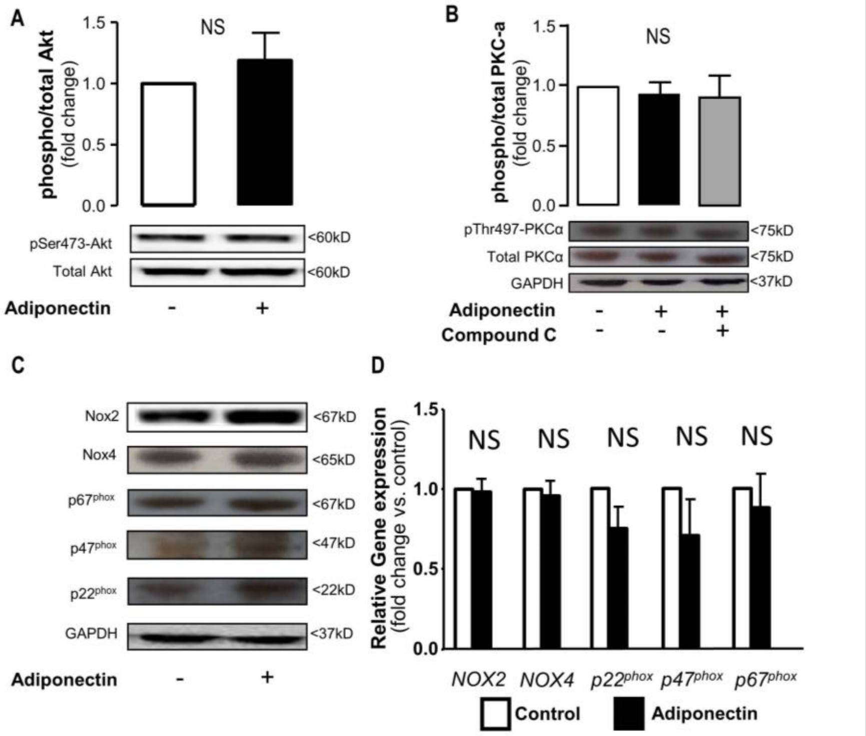

Effects of recombinant adiponectin on the expression of NADPH oxidase subunits and protein kinase C-a phosphorylation. Human myocardium was incubated ex-vivo for 2h in the presence or absence of recombinant human adiponectin (10μg/mL). Adiponectin did not lead to a significant increase in activity of Akt in this tissue, as assessed by Western blotting for phospho-Ser473 Akt, even though a positive trend was observed (Panel A). There was also no effect of adiponectin on the phosphorylation of protein kinase C-α at Thr497 (PKCα, Panel B), suggesting that its effects on NADPH oxidase were independent of any effects on Akt or PKCα signalling. Moreover, adiponectin did not have any effects on the protein levels of Nox2 and Nox4 isoforms or the protein levels of NADPH oxidase subunits, namely $p22^{phox}$, $p47^{phox}$ and $p67^{phox}$ (Panel C-D). n=10-12 for the adiponectin group and n=5-7 for the compound C; NS vs control group.

Online Figure III

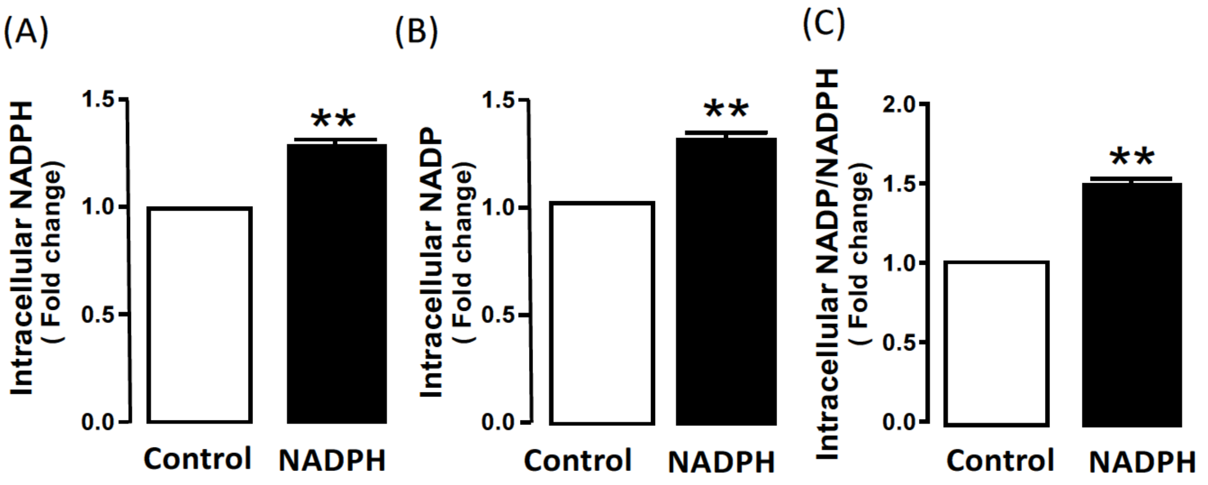

Changes of intracellular NADP/NADPH levels after incubation with exogenous NADPH. H9C2 cells were incubated with / without NADPH 100µmol/L for 18h, in conditions that mimic the co-culture experiments. Then the cells were washed thoroughly and lysed to measure intracellular concentration of NADPH, NADP and total NADP/NADPH using a fluorometric assay. There was a significant increase of both NADPH (by ~25%, A) and NADP (by ~25%, B), with the total NADP/NADPH being increased by ~45% (C) compared to control (**P<0.001 vs control). N=6 independent experiments.

Online Figure IV

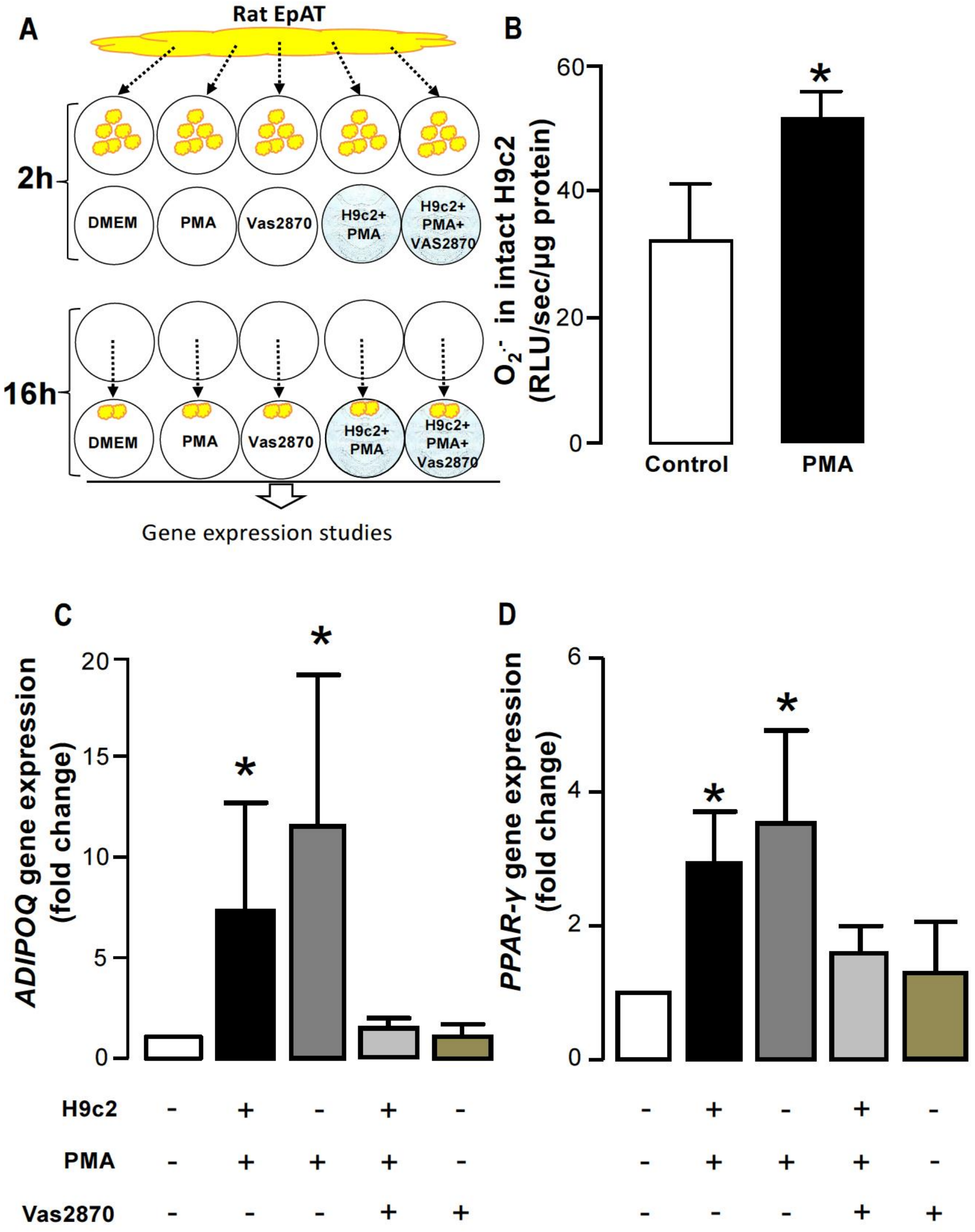

Co-culture of rat epicardial adipose tissue and H9C2 cells pre-stimulated with PMA. To examine whether under conditions of increased endogenous oxidative stress cardiomyocytes release a transferable factor able to affect the activation of PPAR-γ/adiponectin signalling in rat epicardial adipose tissue (EpAT), we exposed H9c2 cells to Phorbol-12-Myristate-13-Acetate (PMA, 160 nmol/L) while rat EpAT was conditioned *ex vivo* (Panel A). After 2h, the rat EpAT was transferred into the H9c2 wells and co-cultured for an additional 16 hours (Panel A). At the end of the incubation period, gene expression of *PPAR-γ* and *ADIPOQ* were studied in the rat EpAT. Addition of PMA to intact H9c2 cells grown on coverslips led to a striking increase of NADPH oxidases-derived superoxide ($O_2^{\cdot-}$, Panel B), as demonstrated by real-time monitoring using lucigenin-enhanced chemiluminescence. Co-incubation of rat EpAT with H9c2 cardiomyocytes stimulated with PMA resulted in an up-regulation of *ADIPOQ* (Panel C) and *PPAR-γ* expression (Panel D) in EpAT at 16h. These effects were prevented by VAS2870 (10nmol/L). However, PMA alone had a similar direct effect on *ADIPOQ* and *PPAR-γ* expression in EpAT even in the absence of H9C2 cells. DMEM: Dulbecco's Modified Eagle Medium; n=7 independent experiments; *p<0.05 vs control group.

Online Figure V

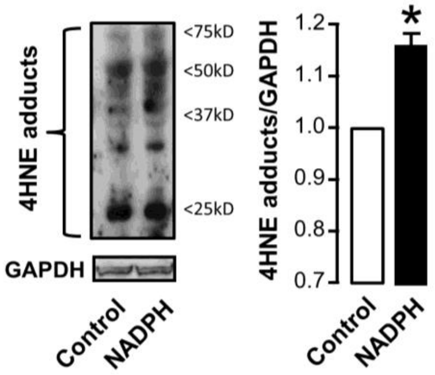

Short-term NADPH stimulation of human myocardium increases the formation of 4-HNE adducts. Human myocardium from patients undergoing coronary artery bypass grafting operation was incubated ex vivo for 16h +/- NADPH 100μmol/L (as a means to induce superoxide generation from NADPH oxidase). This interaction resulted in increased formation of 4-hydroxynonenal protein adducts (by-products of lipid oxidation) in myocardial tissue (Panel A). n=5 independent experiments; *p<0.05 vs control group.

Online Figure VI

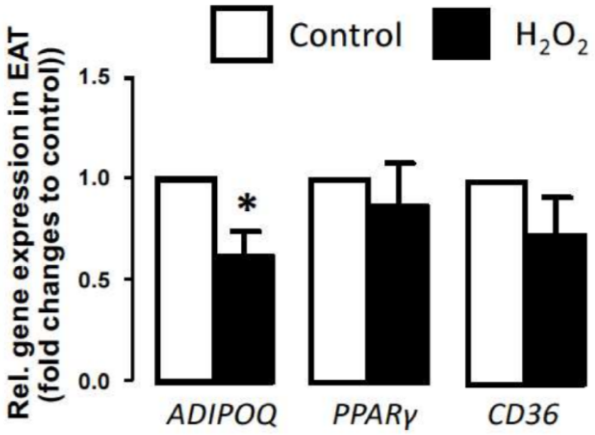

Ex vivo incubations of human epicardial adipose tissue with H_2O_2. Exposure of human epicardial adipose tissue (EAT) to H_2O_2 (100 µmol/L) for 16 h led to a significant reduction in adiponectin (*ADIPOQ*) gene expression, with no significant changes in *PPARγ* or *CD36* expression (n=8 independent experiments, *p<0.05 vs control).

Online Figure VII

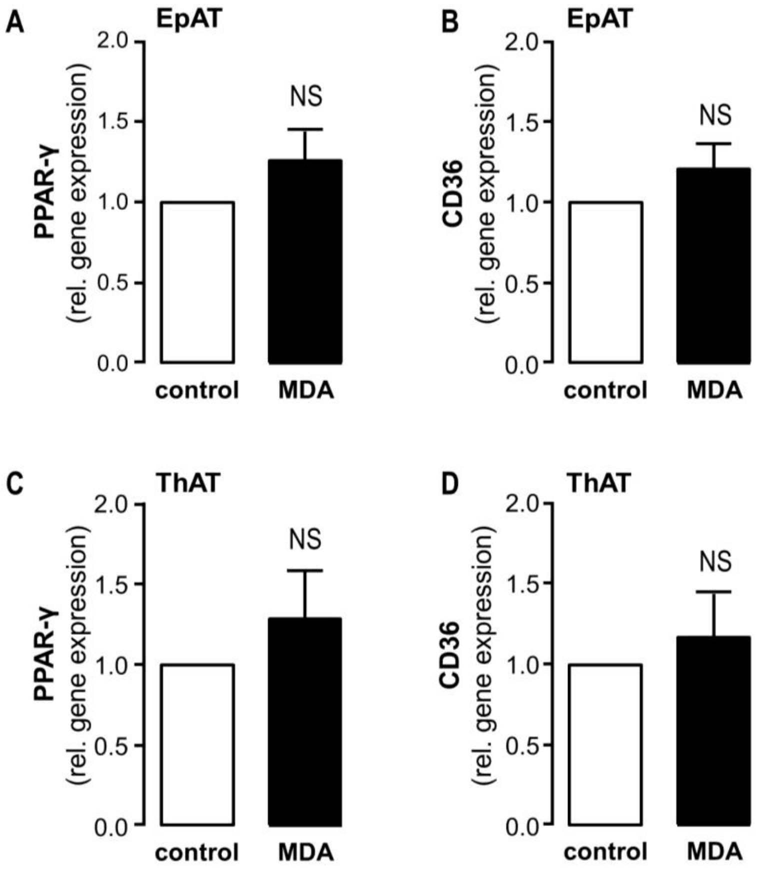

Ex-vivo incubation of epicardial (EpAT) and thoracic (ThAT) adipose tissue with malonyldialdehyde (MDA). EpAT and ThAT from 6 patients undergoing coronary artery bypass grafting was incubated ex-vivo for 16 hours in the presence or absence of MDA 1mM and used for gene expression studies. MDA did not have any effects on the relative expression levels of PPAR-γ and CD36 gene in either EpAT (Panels A-B) or ThAT (Panels C-D). n=5-6 independent experiments; NS: non significant vs control group.

Online Figure VIII

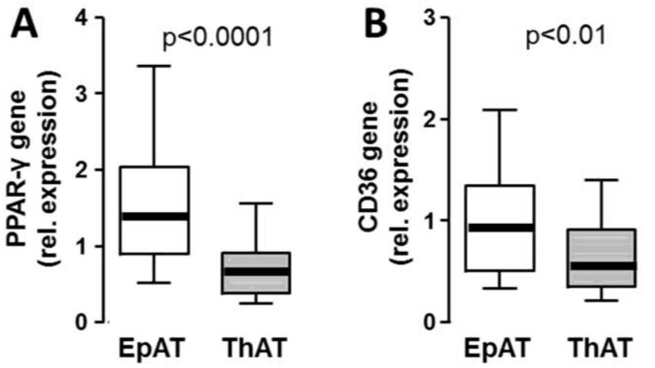

Comparison of peroxisome proliferator activated receptor-γ (PPAR-γ) expression and activation between human adipose tissue depots. Adipose tissue samples of patients with coronary artery disease, were used to compare the expression of PPAR-γ and its downstream mediator CD36 in epicardial (EpAT) vs thoracic adipose tissue (ThAT). Both the expression of PPAR-γ (A) and its downstream molecule CD36 (B) were higher in EpAT compared to ThAT.

Online Figure IX

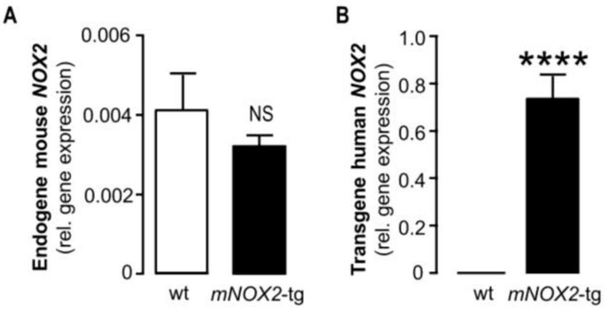

Mouse model of cardiomyocyte-specific overexpression of human *NOX2*. There was no difference in the expression of murine *NOX2* in the heart of *mNOX2*-tg vs the wild type (wt) animals (Panel A). On the contrary, *mNOX2*-tg mice were over-expressing human *NOX2* in their cardiomyocytes, which was not expressed in the wt mice (Panel B). n=9-10 per group; NS: non-significant; ****p<0.0001 vs wt.

Online Figure X

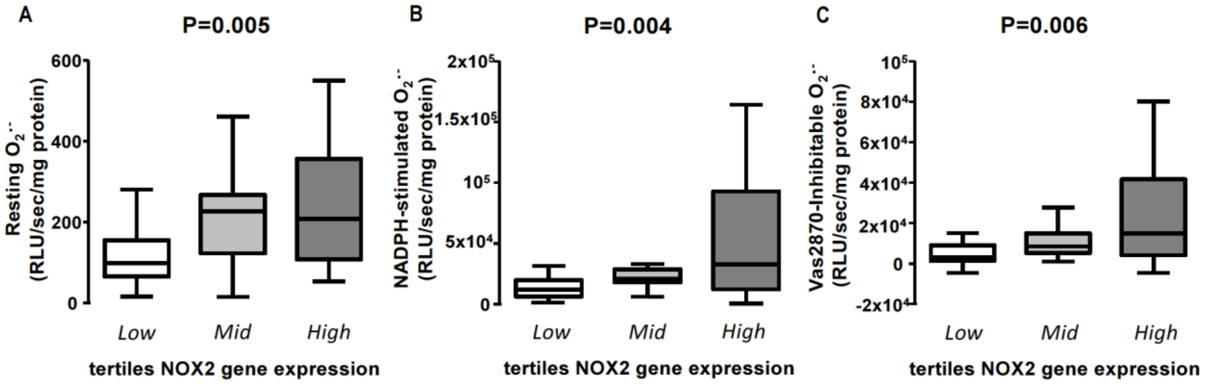

***NOX2* expression and $O_2^{\cdot-}$ generation in the human right atrium appendages (RAA).** High *NOX2* gene expression in human RAA was significantly associated with increased resting $O_2^{\cdot-}$ (A), NADPH-stimulated $O_2^{\cdot-}$ (B) and Vas2870-inhibitable $O_2^{\cdot-}$ (C) in the same tissue.

Online Figure XI

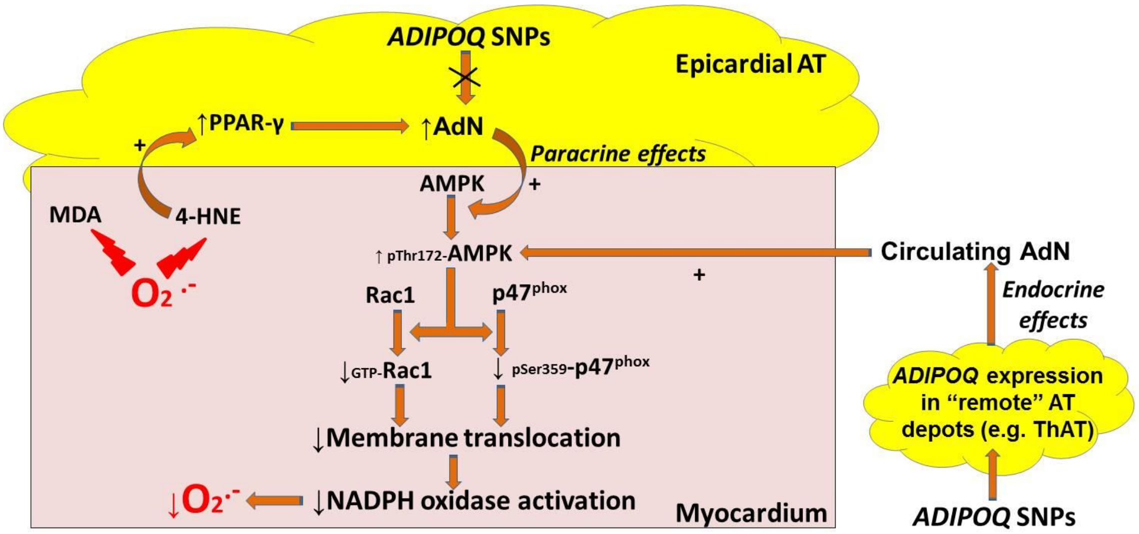

Schematic representation of the bi-directional signalling between epicardial adipose tissue and the myocardium. The cross-talk between epicardial adipose tissue (EpAT) and the myocardium involves the release of oxidation products from the heart (4-hydroxynonenal- 4HNE) able to trigger peroxisome proliferator activated receptor (PPAR)-γ – induced expression of adiponectin (AdN) in EpAT, which may suppress NADPH oxidases activity in the underlying heart muscle in a paracrine way, via an AMPK (AMP kinase) –dependent activation of Rac1 and phosphorylation of p47phox subunits of NADPH oxidase. AT: Adipose Tissue.

Supplemental References

1. Antoniades C, Demosthenous M, Reilly S, Margaritis M, Zhang MH, Antonopoulos A, Marinou K, Nahar K, Jayaram R, Tousoulis D, Bakogiannis C, Sayeed R, Triantafyllou C, Koumallos N, Psarros C, Miliou A, Stefanadis C, Channon KM and Casadei B. Myocardial redox state predicts in-hospital clinical outcome after cardiac surgery effects of short-term pre-operative statin treatment. *J Am Coll Cardiol*. 2012;59:60-70.
2. Wingler K, Altenhoefer SA, Kleikers PW, Radermacher KA, Kleinschnitz C and Schmidt HH. VAS2870 is a pan-NADPH oxidase inhibitor. *Cell Mol Life Sci*. 2012;69:3159-60.
3. Antoniades C, Bakogiannis C, Tousoulis D, Reilly S, Zhang MH, Paschalis A, Antonopoulos AS, Demosthenous M, Miliou A, Psarros C, Marinou K, Sfyras N, Economopoulos G, Casadei B, Channon KM and Stefanadis C. Preoperative atorvastatin treatment in CABG patients rapidly improves vein graft redox state by inhibition of Rac1 and NADPH-oxidase activity. *Circulation*. 2010;122:S66-73.
4. Kim YM, Guzik TJ, Zhang YH, Zhang MH, Kattach H, Ratnatunga C, Pillai R, Channon KM and Casadei B. A myocardial Nox2 containing NAD(P)H oxidase contributes to oxidative stress in human atrial fibrillation. *Circ Res*. 2005;97:629-36.
5. Pfaffl MW. A new mathematical model for relative quantification in real-time RT-PCR. *Nucleic Acids Res*. 2001;29:e45.
6. Brewer AC, Murray TV, Arno M, Zhang M, Anilkumar NP, Mann GE and Shah AM. Nox4 regulates Nrf2 and glutathione redox in cardiomyocytes in vivo. *Free Radic Biol Med*. 2011;51:205-15.
